# Loneliness, depression, and generalized anxiety across eight countries

**DOI:** 10.1007/s00127-025-03029-5

**Published:** 2026-02-05

**Authors:** Salma M. Abdalla, Bernard Banda, Madison Pickerel, Sam B. Rosenberg, Swati Sharma, Sandro Galea

**Affiliations:** 1 Washington University in St Louis School of Public Health, One Brookings Drive, St. Louis, MO 63130 USA; 2 School of Public Health, Boston University, Boston, MA USA

**Keywords:** Loneliness, Depression, Generalized anxiety, Cross-country comparisons

## Abstract

**Background:**

Loneliness is increasingly recognized as a global public health concern linked to adverse mental health outcomes. However, cross-national evidence on its distribution and association with depression and generalized anxiety is limited.

**Methods:**

We analyzed data from the 2023–2024 Global Social Determinants of Health Survey, a cross-sectional, nationally representative survey of 7,997 adults across Brazil, France, India, Indonesia, Nigeria, the Philippines, Türkiye, and the United States. Depression and generalized anxiety were measured using PHQ-9 and GAD-7 screening tools, respectively, while loneliness was self-reported. Weighted bivariate and multivariate (logistic and Poisson regression) models were used to estimate associations between loneliness and mental health outcomes, adjusting for demographic factors.

**Findings:**

Overall, 38.9% of respondents reported loneliness, 9.2% met criteria for depression, and 5.5% for generalized anxiety. Loneliness was more common among younger adults, women, individuals with lower income or education, unmarried individuals, and urban residents. In fully adjusted models, loneliness was associated with depression (OR 2.82 [95% CI: 2.25–3.54]) and generalized anxiety (OR 3.89 [95% CI 2.86–5.28]).

**Interpretation:**

Loneliness is common and strongly associated with depression and generalized anxiety across diverse settings. These findings underscore the importance of integrating strategies that promote social connection into mental health policy and interventions. Future research should explore causal pathways.

**Supplementary Information:**

The online version contains supplementary material available at 10.1007/s00127-025-03029-5.

## Introduction

Mental disorders constitute a significant and increasing proportion of the global burden of morbidity and mortality [1], [2]. Mental disorders accounted for an estimated 80.8 million disability-adjusted life years (DALYs) globally in 1990, a figure that rose to approximately 125.3 million by 2019, according to estimates from the Global Burden of Disease (GBD) study [2]. There is also some evidence that the burden of mental disorders is currently underestimated [3], [4]. Beyond the human and societal impact of poor mental health, the global economic toll of mental disorders was estimated at 5 trillion United States Dollars (USD) [1]. Depressive and anxiety disorders are among the most burdensome mental health disorders worldwide [4]. The recently published 2023 GBD study estimates showed that anxiety disorders saw a sharp rise in burden between 2010–2023, increasing by roughly 60%, followed by depressive disorders with about a 26% rise. In 2023 alone, depressive disorders accounted for an estimated 56 million DALYs, while anxiety disorders contributed roughly 55.4 million [4]. These conditions not only contribute to high levels of disability but also co-occur with other chronic diseases, exacerbating health burdens [5]. The Covid-19 pandemic further amplified the burden of poor mental health [5]. A global analysis across 204 countries and territories estimated that the Covid-19 pandemic contributed to a more than 25% increase in the prevalence of both major depressive disorder and anxiety disorders in 2020, amounting to approximately 53 million and 76 million additional cases, respectively [6].

In parallel, the issue of loneliness is increasingly being understood as a global challenge [7]. Loneliness is often defined as a painful subjective emotional state that arises when there is a discrepancy between desired and actual patterns of social interaction [8]. Recent Organisation for Economic Co-operation and Development (OECD) reports similarly define loneliness as a subjective feeling of being undesirably isolated or of having unmet relational needs [9]. These reports distinguish loneliness from related concepts such as social isolation (objective lack of contact) and social connection (the structure and quality of relationships). Because studies vary in whether they measure subjective loneliness, objective isolation, or broader social connectedness, prevalence estimates and cross-study comparisons can differ [9]. According to a global Gallup survey published in 2024, 23% of people worldwide reported feeling loneliness [10]. The World Health Organization (WHO) declared loneliness a global public health concern in 2023, emphasizing its significant effects on health, well-being, and development and launching a commission to prioritize social connection [11], [12]. Similarly, a 2023 advisory from the United States Surgeon General highlighted the severe health consequences of loneliness, even likening its mortality risk to smoking 15 cigarettes a day [11], [13].

Several international surveys have provided data on loneliness as a public health concern. Studies drawing on the World Health Organization’s Study on Global Ageing and Adult Health (SAGE) [14], [15] have examined loneliness across countries such as China, India, Ghana, Mexico, Russia, and South Africa, describing its prevalence and associations with factors including social support, living arrangements, cognitive function, and psychological distress [16], [17], [18]. European surveys, including Eurobarometer [19] and the Survey of Health, Ageing and Retirement in Europe (SHARE) [20], have generated extensive cross-national estimates of loneliness across European Union (EU) member states and detailed information on loneliness and related health outcomes among adults aged 50 years and older. Together, these studies have helped advance our understanding of loneliness across different regions and demographic groups. Nevertheless, many published analyses focus on single countries, and much of the existing work relies on pre-2020 data, centers on older adults, or examines loneliness separately from standardized assessments of specific mental health disorders such as depression and generalized anxiety.

There is some literature linking loneliness to a number of physical health risk factors (e.g., smoking and obesity) and adverse outcomes (heart disease, stroke, lung disease, and all-cause mortality) [21, 24]. There is also growing literature linking loneliness to adverse mental health outcomes [8, 23]. For example, a longitudinal analysis from the Netherlands showed that loneliness was associated with a poor prognosis of depression among older adults (60–90 years old) at 2-year follow up [25]. A meta-analysis of data published during the Covid-19 pandemic (2020–2022) found a moderate association between symptoms of depression and anxiety [26]. Existing evidence suggests that these relationships may be bidirectional: loneliness can increase vulnerability to depression and anxiety through heightened stress reactivity, reduced social buffering, and other pathways while symptoms of depression and anxiety may, in turn, shape perceptions of social connection and lead to greater feelings of loneliness [23].

Importantly, representative cross-national data describing the relationship between loneliness and adverse mental health outcomes, including depression and anxiety, across diverse demographic and national contexts remain limited. This study aims to (a) describe the prevalence of loneliness, depression, and generalized anxiety across demographic groups and countries; and (b) quantify the relationship between loneliness and both depression and generalized anxiety, when accounting for past diagnosis of adverse mental health outcomes.

## Methods

### Data collection and sample size

This analysis uses data from the Global Social Determinants of Health Survey (GSDS), a cross-sectional survey administered between November 2023 and February 2024. The survey was conducted across eight countries of varying geographic, cultural, linguistic, demographic, and economic composition: Brazil, France, India, Indonesia, Nigeria, the Philippines, Türkiye, and the United States (US). The GSDS was approved by the Boston University Institutional Review Board (H-44020) and committees in specific countries as relevant. De-identified GSDS data and analytic code are available from the authors upon reasonable request.

Adult respondents 18 years and older who resided in one of these eight countries comprised the target population. Ipsos, a global market research and public opinion company, collected data using one of two methods, depending on the country. Surveying conducted in France and the United States used the online KnowledgePanel. In France, Ipsos used dual-frame random-digit dialing of mobile phones and landlines to recruit participants into the KnowledgePanel. In the United States, Ipsos created the KnowledgePanel using address-based sampling. Ipsos employed Computer-Assisted Telephone Interviewing (CATI) through random-digit dialing of mobile phones in the remaining six countries. Respondents did not receive incentives or pay for participation. Respondents were able to refuse or skip any question and could end the survey at any time. Survey mode and language adaptations reflected country context. The questionnaire was translated from English into the primary languages used in each country, including Portuguese, Indonesian, Turkish, English, French, Hausa, Igbo, Yoruba, Filipino, and multiple regional languages in India. Translations were conducted using Ipsos’ standard workflow, which included forward translation, review by local field experts, and final approval prior to fielding to ensure conceptual and linguistic accuracy.

We established a target sample size of at least 1,000 responses in each country. Response rates for CATI countries ranged from 1.1% (Brazil) to 16.7% (India), calculated using American Association for Public Opinion Research (AAPOR) Response Rate 3 (RR3) [27]. Response rates are not reported for the probability-based online panels in France and the United States, as invitations were sent to actively enrolled panel members. Full sampling information and response rates are provided in the Supplementary Methods document. The final sample included 8,298 responses (7,997 were included in this analysis). Supplementary Figure 1 provides an inclusion and exclusion flowchart denoting the sample in each sub-analysis.

### Exposure variables

Exposure variables included age group, gender, educational attainment, income quintile, marital status, area of living (urbanicity), country, loneliness, and past 12-month clinical diagnosis of mental health. Age group was categorical: 18–24, 25–34, 35–44, 45–54, 55 or older. Gender was man or woman. We categorized educational attainment into three levels based on the highest level attained: higher education (college degree or more), secondary education, and primary or no education. For income quintile, we developed five categories for each country based on reported household income: lowest income quintile, second income quintile, middle income quintile, fourth income quintile, or highest income quintile. We measured marital status as married; never married; living with partner; divorced or separated; or widowed. Area of living included: living in a large city; a suburb; a small city or town; or a rural area or village. We also included country as an exposure to account for national contextual differences.

Loneliness was assessed as a key psychosocial exposure, using a single dichotomous self-report item from the Covid-19 and Life Stressors Impact on Mental Health and Well-being (CLIMB) study [28]: “Have you felt alone at any time during the past 12 months?”. Past 12-month clinical diagnosis was measured through self-report items asking whether a doctor, nurse, or other health professional had told the respondent in the past 12 months that they had post-traumatic stress disorder (PTSD), depression, or generalized anxiety.

### Outcome variables

We assessed two mental health outcomes: depression and generalized anxiety. Depression was assessed using the Patient Health Questionnaire-9 (PHQ-9), with a score of 15 or greater as the cutoff to indicate depression. Generalized anxiety was assessed using the Generalized Anxiety Disorder-7 (GAD-7), with a cutoff of 15 or greater. Both tools have been translated into multiple languages and widely validated across cultural contexts [29].

### Statistical analysis

We applied inverse probability weighting to adjust for differential response rates and enhance representativeness of the survey samples. Weights were constructed using a two-step process: (1) design-base weights reflecting each respondent’s probability of selection, and (2) post-stratification calibration to align each country’s sample with national population distributions. In CATI countries, weights were based on gender and age. For France, weights reflected gender by age, education, employment status, and geographic region. In the United States, weights were based on gender by age, race/ethnicity, Census region, metropolitan status, education, and household income. Full weighting details are provided in the Supplementary Methods document. Following data cleaning and the exclusion of respondents with incomplete covariate data, we computed PHQ-9 and GAD-7 sum scores to classify respondents as having depression or generalized anxiety, using established cutoffs of 15 or greater. To assess robustness of this case definition, we also conducted sensitivity analyses using the standard ≥ 10 thresholds for both PHQ-9 and GAD-7.

Because depression and anxiety frequently co-occur, we also examined their joint distribution as an additional descriptive analysis. We created a four-level categorical variable capturing depression only, generalized anxiety only, both conditions, or neither. In this sample, 3.6% (N = 315) met criteria for both disorders, indicating meaningful comorbidity; however, because this subgroup was relatively small, comorbidity was not modeled separately. Descriptive results are reported in the Supplementary Methods document.

We then conducted bivariate analyses to examine the association between demographic characteristics and loneliness with each mental health outcome. We reported unweighted frequencies and weighted percentages with 95% confidence intervals, accounting for complex survey design using Rao–Scott adjusted chi-square tests [30]. To examine associations between demographics, loneliness, past 12-month clinical diagnosis of PTSD, depression, or generalized anxiety, and mental health outcomes, we fitted both weighted logistic and Poisson regression models. Logistic regression models estimated odds ratios (ORs) and 95% confidence intervals (CIs) for depression and generalized anxiety, while Poisson regression models estimated prevalence ratios (PRs) and 95% CIs for PHQ-9 and GAD-7 summed scores. We used Poisson regression with design-based robust standard errors, as implemented in the survey package (svyglm). This approach is recommended for complex survey data because the sandwich (robust) estimator provides valid inference even when the Poisson mean-variance assumption is violated, including in the presence of overdispersion [31], [32].

Three logistic regression models were specified for each outcome: Model 1 estimated unadjusted associations between each covariate and the outcome; Model 2 adjusted for all demographic covariates (age group, gender, educational attainment, income quintile, marital status, area of living, and country) and loneliness, but excluded past 12-month clinical diagnosis of an adverse mental health outcome; and Model 3 included all demographic covariates and past 12-month clinical diagnosis of an adverse mental health outcome in the fully adjusted model (see Supplementary Methods document for detailed model specification). To assess the robustness of our models, we conducted multicollinearity diagnostics and model-fit evaluations. Variance inflation factors (VIFs) in the fully adjusted models were low (1.02–2.18), indicating no meaningful multicollinearity. Model fit was evaluated using design-based likelihood ratio tests, which showed that adding loneliness and past 12-month diagnosis significantly improved the fit of both depression and generalized anxiety models; full diagnostic results are provided in Supplementary Methods document. All analyses were conducted in R (version 4.4.3) using the data.table, tidyverse, survey, srvyr, and gtsummary packages[33–39]

## Results

### Sample characteristics

Table 1 shows sample characteristics. Of the 8,298 respondents, we included 7,997 (96.4%) respondents with complete data for age group, gender, educational attainment, and loneliness in this analysis (Supplementary Figure 1). Age distribution was mostly balanced, with the largest share aged 55 or older (25.7% [95% CI: 24.4%, 27.0%]), and the smallest group aged 45–54 (15.0% [95% CI: 14.1%, 15.9%]). Respondents were largely evenly distributed by gender, with 49.5% [95% CI: 48.1%, 50.8%] identifying as men and 50.5% [95% CI: 49.2%, 51.9%] as women. In terms of educational attainment, 29.8% [95% CI: 28.6%, 30.9%] reported higher education (college degree or more), 47.6% [95% CI: 46.3%, 48.9%] reported secondary education, and 22.6% [95% CI: 21.5%, 23.8%] reported primary or no education. Respondents were largely evenly distributed across income quintiles, with the highest comprising 22.1% [95% CI: 20.8%, 23.3%], middle comprising 18.0% [95% CI: 16.9%, 19.1%], and lowest income quintile comprising 19.9% [95% CI: 18.7%, 21.1%]. In terms of marital status, just over half of respondents were married (53.0% [95% CI: 51.7%, 54.4%]), followed by those who had never married (27.8% [95% CI: 26.7%, 29.0%]), were living with a partner (7.9% [95% CI: 7.2%, 8.6%]), were divorced or separated (6.9% [95% CI: 6.2%, 7.5%]), or widowed (4.4% [95% CI: 3.8%, 5.1%]). In terms of area of residence, 38.2% [95% CI: 37.0%, 39.5%] lived in a large city, while 24.2% [95% CI: 23.1%, 25.3%] resided in a rural area or village. The remaining respondents lived in suburbs (15.0% [95% CI: 14.1%, 16.0%]) or small towns (22.6% [95% CI: 21.5%, 23.7%]). Respondents were relatively evenly distributed across the eight countries, with the largest shares from France (13.4% [95% CI: 12.4%, 14.3%]) and Türkiye (13.3% [95% CI: 12.4%, 14.1%]), and the smallest from the Philippines (11.7% [95% CI: 10.9%, 12.6%]) and the United States (11.9% [95% CI: 11.2%, 12.7%]).

**Table 1:** Sample demographics

Characteristic	Unweighted N = 7997	Weighted % [95% CI]
**Age group**		
18–24	1337	16.5% [15.5%, 17.4%]
25–34	2074	21.8% [20.8%, 22.7%]
35–44	1780	21.1% [20.1%, 22.2%]
45–54	1288	15.0% [14.1%, 15.9%]
55 or older	1518	25.7% [24.4%, 27.0%]
**Gender**		
Man	4216	49.5% [48.1%, 50.8%]
Woman	3781	50.5% [49.2%, 51.9%]
**Educational attainment**		
Higher education	2530	29.8% [28.6%, 30.9%]
Secondary education	3870	47.6% [46.3%, 48.9%]
Primary or no education	1597	22.6% [21.5%, 23.8%]
**Income quintile**		
Highest quintile	1382	22.1% [20.8%, 23.3%]
Fourth quintile	1366	20.8% [19.6%, 22.0%]
Middle quintile	1190	18.0% [16.9%, 19.1%]
Second quintile	1283	19.3% [18.2%, 20.4%]
Lowest quintile	1189	19.9% [18.7%, 21.1%]
**Marital status**		
Married	4188	53.0% [51.7%, 54.4%]
Never married	2345	27.8% [26.7%, 29.0%]
Living with partner	610	7.9% [7.2%, 8.6%]
Divorced or separated	531	6.9% [6.2%, 7.5%]
Widowed	283	4.4% [3.8%, 5.1%]
**Urbanicity**		
Large city	3000	38.2% [37.0%, 39.5%]
Suburb near a large city	1164	15.0% [14.1%, 16.0%]
Small city or town	1833	22.6% [21.5%, 23.7%]
Rural area or village	1982	24.2% [23.1%, 25.3%]
**Country**		
United States	954	11.9% [11.2%, 12.7%]
Brazil	985	12.3% [11.6%, 13.2%]
France	1062	13.4% [12.4%, 14.3%]
India	969	12.0% [11.1%, 12.9%]
Indonesia	1011	12.7% [11.8%, 13.6%]
Nigeria	1021	12.8% [11.9%, 13.7%]
The Philippines	938	11.7% [10.9%, 12.6%]
Türkiye	1057	13.3% [12.4%, 14.1%]
**Loneliness status**		
No	4856	61.1% [59.8%, 62.4%]
Yes	3141	38.9% [37.6%, 40.2%]
**Depression**		
No	7241	90.8% [90.0%, 91.5%]
Yes	756	9.2% [8.5%, 10.0%]
**Generalized anxiety**		
No	7540	94.5% [94.0%, 95.1%]
Yes	457	5.5% [4.9%, 6.0%]
**Past 12-month clinical diagnosis** ^1^		
No	6335	80.3% [79.2%, 81.3%]
Yes	579	19.7% [18.7%, 20.8%]
**Comorbid depression & generalized anxiety**		
No comorbidity	7682	96.4% [95.9%, 96.8%]
Comorbid depression & generalized anxiety	315	3.6% [3.2%, 4.1%]

*Estimates and confidence intervals weighted using inverse probability weights. Confidence intervals calculated with Rao-Scott correction. The sample was weighted using gender and age in CATI countries; gender by age, education, employment, and region in France; and gender by age, race/ethnicity, Census region, metropolitan status, education, and income in the US. Missing data: income quintile (N* = *1,587), marital status (N* = *40), urbanicity (N* = *18).*^*1*^*The variable past 12-month clinical diagnosis included a clinical diagnosis of post-traumatic stress disorder, depression, or generalized anxiety within the past 12 months*

The overall prevalence of depression was 9.2% [95% CI: 8.5%, 10.0%] and of generalized anxiety was 5.5% [95% CI: 4.9%, 6.0%]. 19.7% [95% CI: 18.7%, 20.8%] of all respondents reported a clinical diagnosis of either PTSD, depression, or generalized anxiety in the past 12 months. The overall prevalence for respondents reporting comorbid depression and generalized anxiety was 3.6% [95% CI: 3.2%, 4.1%]). 

Sensitivity analyses using the standard PHQ-9 ≥ 10 and GAD-7 ≥ 10 thresholds produced the expected increase in symptom prevalence but did not alter the main findings. The weighted prevalence of depression increased from 9.2% (≥ 15) to 20.0% (≥ 10). For generalized anxiety, applying the ≥ 10 cutoff similarly increased case identification. Sensitivity analyses prevalence estimates with confidence intervals are presented in the Supplementary Methods document.

### Loneliness prevalence by demographic factors

Supplementary Table 1 presents the distribution of self-reported loneliness across demographic characteristics. Overall, 38.9% [95% CI: 37.6%, 40.2%] of respondents reported feeling lonely. Loneliness was most prevalent among younger adults, decreasing steadily with age from 46.8% [95% CI: 43.7%, 50.0%] among those aged 18–24 to 30.4% [95% CI: 27.5%, 33.3%] among those aged 55 or older. Women more often reported loneliness than men (41.1% [95% CI: 39.2%, 42.9%] vs. (36.6% [95% CI: 34.9%, 38.4%]). Respondents with secondary education had the highest prevalence of loneliness (42.3% [95% CI: 40.5%, 44.2%]), while those with primary or no education had the lowest (33.4% [95% CI: 30.7%, 36.2%]). Loneliness increased as income decreased, from 34.0% [95% CI: 31.0%, 37.1%] in the highest income quintile to 48.1% [95% CI: 44.7%, 51.4%] in the lowest. Only 29.5% [95% CI: 27.8%, 31.1%] of married respondents reported loneliness compared to 53.6% [95% CI: 48.6%, 58.6%] of those divorced or separated and 50.2% [95% CI: 47.8%, 52.5%] of those never married. Loneliness was highest in large cities (41.4% [95% CI: 39.3%, 43.5%]) and lowest in rural areas (36.0% [95% CI: 33.4%, 38.6%]). Countries with the highest loneliness prevalence were the Philippines (48.9% [95% CI: 45.0%, 52.8%]), Brazil (47.5% [95% CI: 44.1%, 50.9%]), and Nigeria (45.8% [95% CI: 42.0%, 49.7%]), Conversely, India, reported comparatively low loneliness prevalence (19.2% [95% CI: 16.0%, 22.3%]). Among those who responded yes to a past 12-month clinical diagnosis of PTSD, depression, or anxiety, 64.7% [95% CI: 61.9%, 67.5%] reported feeling lonely compared to 32.5% [95% CI: 31.1%, 33.8%] of those who did not report a clinical diagnosis of PTSD, depression, or anxiety in the past 12 months.

### Depression prevalence by demographics and loneliness status

Table 2 shows depression by different demographic variables and past 12-month clinical diagnosis of PTSD, depression, or generalized anxiety. Overall, 9.2% [95% CI: 8.5%, 10.0%] of respondents reported having depression. Depression decreased with age, from 13.4% [95% CI: 11.4%, 15.5%] in the 18–24 age group to 5.5% [95% CI: 4.2%, 7.1%] in the 55 or older age group. Women reported higher depression (11.3% [95% CI: 10.2%, 12.5%]) than men (7.1% [95% CI: 6.3%, 8.1%]). Those with secondary education had the highest prevalence of depression at 10.6% [95% CI: 9.6%, 11.8%], followed by respondents with primary or no education (9.5% [95% CI: 7.9%, 11.2%]) and higher education (6.8% [95% CI: 5.7%, 8.0%]). The prevalence of depression increased with decreasing income quintile, from 6.9% [95% CI: 5.4%, 8.6%] in the highest income quintile to 14.9% [95% CI: 12.7%, 17.4%] in the lowest income quintile. Married respondents had the lowest (6.7% [95% CI: 5.8%, 7.6%]) while respondents who had never married had the highest prevalence of depression (12.8% [95% CI: 11.3%, 14.5%]). The prevalence of depression was similar across areas of living ranging from 8.4% [95% CI: 6.9%, 9.9%] in a rural area or village to 11.3% [95% CI: 9.7%, 13.1%] in a small city or town. The prevalence of depression ranged from 3.4% [95% CI: 2.1%, 5.1%] in India to 15.8% [95% CI: 13.5%, 18.3%] in Brazil. Finally, the prevalence of depression among those that reported a clinical diagnosis of PTSD, depression, or generalized anxiety in the past 12 months was 27.6% [95% CI: 25.0%, 30.2%] compared to 4.7% [95% CI: 4.1%, 5.3%] of those who did not report a past clinical diagnosis.

**Table 2:** Depression and generalized anxiety prevalence stratified by demographics and loneliness status

Characteristic	Depression		Generalized anxiety	
	Unweighted N = 756	Prevalence [95% CI]	Unweighted N = 457	Prevalence [95% CI]
**Age group**				
18–24	173	13.4% [11.4%, 15.5%]	111	8.4% [6.8%, 10.1%]
25–34	242	11.4% [9.9%, 13%]	134	6.6% [5.5%, 7.9%]
35–44	152	9.0% [7.5%, 10.6%]	100	5.3% [4.3%, 6.5%]
45–54	110	8.2% [6.7%, 10.0%]	67	4.8% [3.6%, 6.2%]
55 or older	79	5.5% [4.2%, 7.1%]	45	3.1% [2.2%, 4.3%]
**Gender**				
Man	311	7.1% [6.3%, 8.1%]	156	3.6% [3.0%, 4.3%]
Woman	445	11.3% [10.2%, 12.5%]	301	7.3% [6.4%, 8.2%]
**Educational attainment**				
Higher education	178	6.8% [5.7%, 8.0%]	90	3.7% [2.9%, 4.7%]
Secondary education	420	10.6% [9.6%, 11.8%]	257	6.2% [5.4%, 7.1%]
Primary or no education	158	9.5% [7.9%, 11.2%]	110	6.1% [4.9%, 7.5%]
**Income quintile**				
Highest quintile	93	6.9% [5.4%, 8.6%]	55	4.3% [3.1%, 5.8%]
Fourth quintile	95	6.6% [5.3%, 8.2%]	49	3.1% [2.3%, 4.1%]
Middle quintile	139	10.0% [8.3%, 12%]	79	5.8% [4.5%, 7.2%]
Second quintile	141	10.7% [8.9%, 12.6%]	89	7.0% [5.5%, 8.6%]
Lowest quintile	176	14.9% [12.7%, 17.4%]	114	9.1% [7.4%, 11.1%]
**Marital status**				
Married	288	6.7% [5.8%, 7.6%]	168	3.6% [3.0%, 4.3%]
Never married	299	12.8% [11.3%, 14.5%]	174	7.7% [6.5%, 9.1%]
Living with partner	76	11.8% [9.2%, 14.8%]	60	9.4% [7.0%, 12.2%]
Divorced or separated	66	12.1% [9.2%, 15.5%]	42	7.2% [5.1%, 9.8%]
Widowed	25	8.0% [4.8%, 12.2%]	12	4.0% [1.9%, 7.1%]
**Urbanicity**				
Large city	274	8.7% [7.6%, 9.8%]	152	4.4% [3.7%, 5.2%]
Suburb near a large city	110	9.1% [7.3%, 11.2%]	76	6.4% [4.9%, 8.2%]
Small city or town	212	11.3% [9.7%, 13.1%]	132	7.1% [5.8%, 8.5%]
Rural area or village	160	8.4% [6.9%, 9.9%]	97	5.2% [4.1%, 6.4%]
**Country**				
United States	67	7.2% [5.6%, 9.0%]	44	4.6% [3.4%, 6.2%]
Brazil	162	15.8% [13.5%, 18.3%]	139	12.9% [10.8%, 15.1%]
France	96	10.9% [8.5%, 13.6%]	44	4.8% [3.3%, 6.7%]
India	33	3.4% [2.1%, 5.1%]	15	1.5% [0.8%, 2.6%]
Indonesia	62	5.7% [4.2%, 7.4%]	41	3.9% [2.7%, 5.4%]
Nigeria	93	7.1% [5.6%, 8.9%]	38	3.2% [2.1%, 4.5%]
The Philippines	119	12.1% [9.7%, 14.7%]	75	7.6% [5.8%, 9.8%]
Türkiye	124	11.5% [9.5%, 13.8%]	61	5.3% [4.0%, 6.9%]
**Loneliness status**				
No	216	4.1% [3.5%, 4.7%]	103	1.8% [1.5%, 2.2%]
Yes	540	17.3% [15.8%, 18.9%]	354	11.2% [10.0%, 12.5%]
**Past 12-month clinical diagnosis** ^1^				
No	321	4.7% [4.1%, 5.3%]	183	2.7% [2.2%, 3.1%]
Yes	426	27.6% [25.0%, 30.2%]	266	16.5% [14.4%, 18.6%]

*Estimates and confidence intervals weighted using inverse probability weights. Confidence intervals calculated with Rao-Scott correction. The sample was weighted using gender and age in CATI countries; gender by age, education, employment, and region in France; and gender by age, race/ethnicity, Census region, metropolitan status, education, and income in the US. For depression, missing data: income quintile (N* = *112), marital status (N* = *2), past 12-month clinical diagnosis (N* = *10). Missing data for generalized anxiety: income quintile (N* = *71), marital status (N* = *1), past 12-month clinical diagnosis. The variable past 12-month clinical diagnosis included a clinical diagnosis of post-traumatic stress disorder, depression, or generalized anxiety within the past 12 months*

**Fig. 1:**
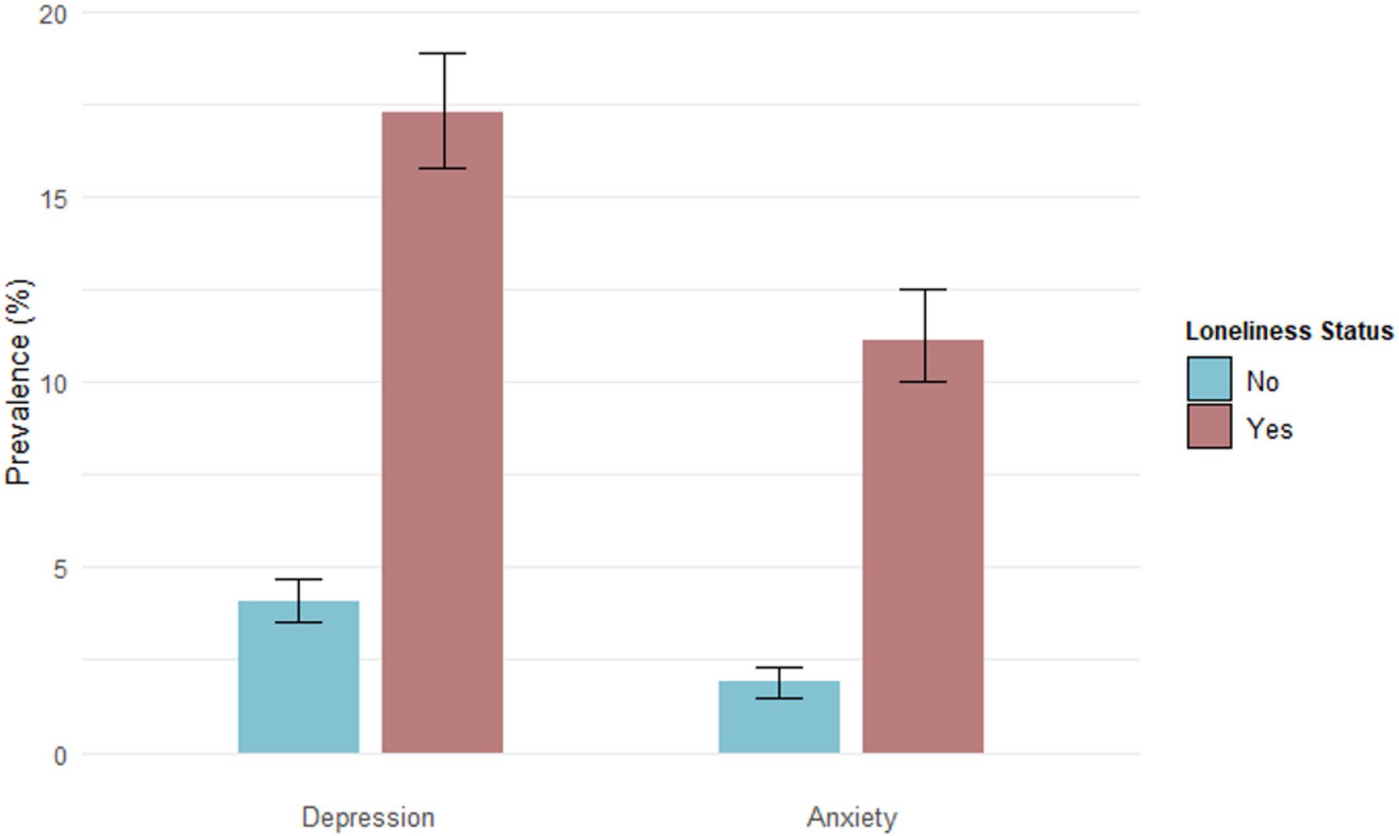
Prevalence of depression and generalized anxiety stratified by loneliness status

**Fig. 2:**
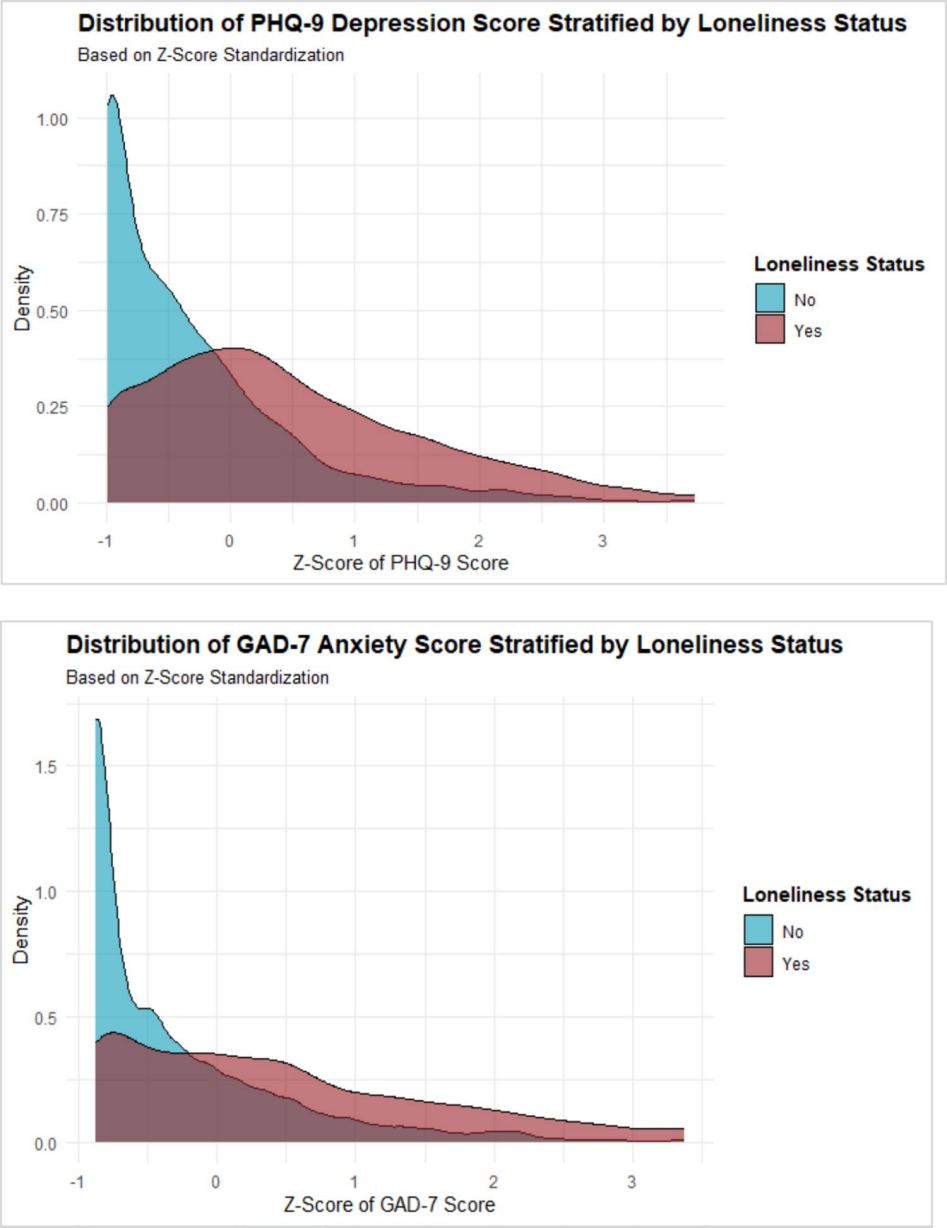
**A** **and B** Distribution of depression and generalized anxiety stratified by loneliness status with Z-score standardization

Figure 1 shows the prevalence of depression by loneliness status. Among those who reported loneliness, 540 individuals (17.3% [95% CI: 15.8%, 18.9%]) met the threshold for depression and among those who do not report loneliness, 216 individuals (4.1% [95% CI: 3.5%, 4.7%]) met the threshold for depression. Figure 2A illustrates the distribution of standardized PHQ-9 depression scores by loneliness status. Individuals who reported loneliness had consistently higher PHQ-9 scores, with their density curve shifted rightward compared to those who did not report loneliness. The peak of the distribution for respondents who did not report loneliness was concentrated at lower Z-scores (closer to negative one), while who reported loneliness showed a broader spread towards higher Z-scores, indicating greater depression symptom severity. In absolute terms, among respondents who were lonely, only 8.5% [95% CI: 7.2%, 9.7%] reported no depression symptoms (scored zero on the PHQ-9 screening) compared to 29.8% [95% CI: 28.2%, 31.4%] of respondents who were not lonely.

### Odds ratios of depression by demographics and loneliness status

Table 3 shows the odds ratios of depression across unadjusted models and adjusted models for all variables. This section highlights statistically significant results from Model 3, which included all demographic variables, loneliness, and past 12-month clinical diagnosis of PTSD, depression, or generalized anxiety unless otherwise indicated. Respondents in the 35–44 age group (OR: 0.54 [95% CI: 0.37, 0.78]), 45–54 age group (OR: 0.47 [95% CI: 0.31, 0.69]), and the 55 or older age group (OR: 0.29 [95% CI: 0.18, 0.48]) all had reduced odds of depression compared to those in the 18–24 age group. Women had 1.30 [95% CI: 1.04, 1.61] times the odds of depression compared to men. Respondents with secondary education had 1.58 [95% CI: 1.21, 2.06] times the odds, and respondents with primary or no education had 1.92 [95% CI: 1.34, 2.74] times the odds of depression compared to those with higher education. While individuals in the different income quintiles initially had significantly higher odds of depression compared to the highest income group, these associations attenuated and lost statistical significance after adjusting for all covariates in Model 3, with the exception of the lowest quintile (OR: 1.51 [95% CI: 1.06, 2.15]), which remained significantly associated with greater odds of depression. In terms of marital status, individuals who were never married, living with a partner, or divorced or separated, had significantly higher odds of depression in the unadjusted model, however, these associations were no longer statistically significant after adjusting for demographic covariates and loneliness in Model 2 and for past 12-month clinical diagnosis of PTSD, depression, or generalized anxiety in the fully adjusted Model 3.

**Table 3:** Logistic regression of depression by sample demographics, loneliness status, and past 12-month clinical diagnosis

Characteristic	Model 1: Unadjusted OR [95% CI]	Model 2: Adjusted OR excluding past 12-month clinical diagnosis [95% CI]	Model 3: Adjusted OR including past 12-month clinical diagnosis [95% CI]
**Age group**			
18–24	Reference	Reference	Reference
25–34	0.83 [0.66, 1.05]	0.81 [0.61, 1.08]	0.74 [0.54, 1.00]
35–44	0.64 [0.49, 0.83]***	0.58 [0.41, 0.82]**	0.54 [0.37, 0.78]**
45–54	0.58 [0.44, 0.77]***	0.55 [0.38, 0.80]**	0.47 [0.31, 0.69]***
55 or older	0.38 [0.27, 0.53]***	0.32 [0.20, 0.52]***	0.29 [0.18, 0.48]***
**Gender**			
Man	Reference	Reference	Reference
Woman	1.66 [1.39, 1.98]***	1.59 [1.30, 1.95]***	1.30 [1.04, 1.61]*
**Educational attainment**			
Higher education	Reference	Reference	Reference
Secondary education	1.63 [1.32, 2.02]***	1.52 [1.18, 1.95]**	1.58 [1.21, 2.06]***
Primary or no education	1.43 [1.10, 1.87]**	1.81 [1.29, 2.55]***	1.92 [1.34, 2.74]***
**Income quintile**			
Highest quintile	Reference	Reference	Reference
Fourth quintile	0.96 [0.68, 1.36]	0.87 [0.61, 1.23]	0.89 [0.61, 1.29]
Middle quintile	1.51 [1.09, 2.09]*	1.11 [0.80, 1.54]	1.16 [0.83, 1.63]
Second quintile	1.62 [1.18, 2.22]**	1.11 [0.80, 1.55]	1.15 [0.81, 1.63]
Lowest quintile	2.38 [1.74, 3.25]***	1.51 [1.08, 2.12]*	1.51 [1.06, 2.15]*
**Marital status**			
Married	Reference	Reference	Reference
Never married	2.06 [1.68, 2.51]***	1.05 [0.79, 1.38]	0.96 [0.72, 1.28]
Living with partner	1.87 [1.38, 2.54]***	0.89 [0.62, 1.27]	0.79 [0.55, 1.14]
Divorced or separated	1.92 [1.38, 2.67]***	0.96 [0.66, 1.41]	0.84 [0.57, 1.23]
Widowed	1.21 [0.72, 2.05]	0.85 [0.43, 1.68]	0.85 [0.42, 1.72]
**Urbanicity**			
Large city	Reference	Reference	Reference
Suburb near a large city	1.06 [0.81, 1.39]	1.39 [1.01, 1.93]*	1.50 [1.07, 2.10]*
Small city or town	1.35 [1.08, 1.68]**	1.39 [1.08, 1.78]*	1.39 [1.06, 1.83]*
Rural area or village	0.96 [0.76, 1.23]	1.23 [0.92, 1.65]	1.33 [0.98, 1.81]
**Country**			
United States	Reference	Reference	Reference
Brazil	2.42 [1.77, 3.32]***	1.98 [1.39, 2.83]***	1.85 [1.28, 2.70]**
France	1.58 [1.10, 2.28]*	1.69 [1.12, 2.56]*	1.73 [1.12, 2.65]*
India	0.45 [0.27, 0.77]**	0.47 [0.24, 0.93]*	0.47 [0.23, 0.95]*
Indonesia	0.78 [0.52, 1.15]	0.49 [0.31, 0.80]**	0.63 [0.39, 1.04]
Nigeria	0.99 [0.69, 1.42]	1.04 [0.69, 1.58]	1.26 [0.82, 1.92]
The Philippines	1.77 [1.25, 2.51]**	1.28 [0.85, 1.93]	1.72 [1.12, 2.65]*
Türkiye	1.68 [1.20, 2.34]**	1.59 [1.08, 2.34]*	2.02 [1.33, 3.05]***
**Loneliness status**			
No	Reference	Reference	Reference
Yes	4.94 [4.06, 6.00]***	4.07 [3.29, 5.04]***	2.82 [2.25, 3.54]***
**Past 12-month clinical diagnosis** ^1^			
No	Reference	—	Reference
Yes	7.70 [6.41, 9.26]***	—	5.43 [4.35, 6.78]***

* p* < *0.05; p* < *0.01; p* < *0.001. The sample was weighted with inverse probability weights for gender and age in CATI countries; gender by age, level of education, employment status, and geographic region in France; and gender by age, race/ethnicity, Census region, metropolitan status, education, and household income in the United States. The models removed 1,587 respondents with missing income, 31 respondents with missing marital status, and 16 respondents with missing urbanicity. Overall, the model removed 1,634 respondents. CI* = *Confidence Interval, OR* = *Odds Ratio.* *Model 1 includes depression and unadjusted covariates (age group, gender, educational attainment, income quintile, marital status, urbanicity, country, loneliness status, and past 12-month clinical diagnosis). Model 2 includes all covariates except past 12-month clinical diagnosis. Model 3 includes all covariates (age group, gender, educational attainment, income quintile, marital status, urbanicity, country, loneliness status, and past 12-month clinical diagnosis.) *^1^*Past 12-month clinical diagnosis includes diagnosis of post-traumatic stress disorder, depression, or generalized anxiety*

Compared to respondents living in a large city, living in a suburb (OR: 1.50 [95% CI: 1.07, 2.10]) or a small city or town (OR: 1.39 [95% CI: 1.06, 1.83]) was associated with significantly higher odds of depression. However, living in a rural area or village was not significantly associated with depression in any model. For country, compared to the United States, respondents living in Brazil (OR: 1.85 [95% CI: 1.28, 2.70]), France (OR: 1.73 [95% CI: 1.12, 2.65]), the Philippines (OR: 1.72 [95% CI: 1.12, 2.65]), and Türkiye (OR: 2.02 [95% CI: 1.33, 3.05]) had higher odds of depression. In contrast, respondents in India (OR: 0.47 [95% CI: 0.23, 0.95]) had lower odds of depression. Respondents who reported feeling lonely had significantly higher odds of depression compared to those who did not report loneliness, with an odds ratio of 2.82 [95% CI: 2.25, 3.54]. Reporting a clinical diagnosis of PTSD, depression, or generalized anxiety in the past 12 months was associated with over five times the odds of depression (OR: 5.43 [95% CI: 4.35, 6.78]).

The sensitivity analysis using the PHQ-9 ≥ 10 threshold produced results that closely mirrored those from the ≥ 15 threshold, with the direction and relative magnitude of associations largely unchanged. As expected with a lower cutoff, several estimates shifted modestly toward the null, while a few groups—such as those living in rural areas, and certain countries—increased significance or met significance only under the ≥ 10 threshold. Overall, most predictors remained strong and consistent across both specifications. Full sensitivity analysis results for depression can be seen in Supplementary Table 2.

In addition to logistic regression, we performed Poisson regression using the PHQ-9 sum scores to assess the association between depression symptoms and demographic variables. Results from the Poisson models largely mirrored the findings from the logistic regression models. After adjusting for demographic characteristics, loneliness, and past 12-month clinical diagnosis (Model 3), the relative difference by all demographic variables remained consistent but attenuated with a few exceptions. The association between living in a small town and depression was no longer significant. The association between living in Nigeria (PR: 1.26 [95% CI: 1.14, 1.40]) and depression symptoms became significant. Respondents who reported feeling lonely continued to have significantly higher depression symptoms (PR: 1.69 [95% CI: 1.60, 1.78]) compared to those who did not report loneliness. Full Poisson regression results for depression are presented in Supplementary Table 3.

### Generalized anxiety prevalence by demographics and loneliness status

Table 2 shows generalized anxiety along different demographic variables. Prevalence decreased with increasing age group, from 8.4% [95% CI: 6.8%, 10.1%] in the 18–24 age group to 3.1% [95% CI: 2.2%, 4.3%] in the 55 or older age group. 7.3% [95% CI: 6.4%, 8.2%] of women and 3.6 [95% CI: 3.0%, 4.3%] of men had generalized anxiety. Respondents with primary or no education had the highest prevalence of generalized anxiety (6.1% [95% CI: 4.9%, 7.5%] and respondents with higher education had the lowest (3.7% [95% CI: 2.9%, 4.7%]). The prevalence was lowest in the fourth income quintile (3.1% [95% CI: 2.3%, 4.1%]), and highest in the lowest income quintile (9.1% [95% CI: 7.4%, 11.1%]). Married respondents had the lowest prevalence (3.6% [95% CI: 3.0%, 4.3%]), while respondents living with partner had the highest (9.4% [95% CI: 7.0%, 12.2%]). Generalized anxiety prevalence was similar across area of living, ranging from 4.4% [95% CI: 3.7%, 5.2%] for those living in a large city to 7.1% [95% CI: 5.8%, 8.5%] for those living in a small city or town. Across countries, India had the lowest prevalence of generalized anxiety (1.5% [95% CI: 0.8%, 2.6%]), while Brazil had the highest (12.9% [95% CI: 10.8%, 15.1%]). Finally, the prevalence of generalized anxiety among those that reported a clinical diagnosis of either PTSD, depression, or generalized anxiety in the past 12 months was 16.5% [95% CI: 14.4%, 18.6%] compared to 2.7% [95% CI: 2.2%, 3.1%] of those who did not report a clinical diagnosis.

Figure 1 shows the prevalence of generalized anxiety by loneliness status. Among those who reported loneliness, 11.2% [95% CI: 10.0%, 12.5%] met the criteria for generalized anxiety, compared to 1.8% [95% CI: 1.5%, 2.2%] among those who did not report loneliness. Figure 2B displays the distribution of standardized GAD-7 anxiety scores stratified by loneliness status. Individuals who reported loneliness had consistently higher GAD-7 scores, with their density curve shifted to the right compared to those who did not report loneliness. Respondents who did not report loneliness had a peak at lower Z-scores (around negative one), whereas those who reported loneliness had a broader distribution towards higher Z-scores, reflecting greater anxiety symptom severity. In absolute terms, among respondents who were lonely, only 15.8% [95% CI: 14.2%, 17.5%] reported no generalized anxiety symptoms compared to 41.1% [95% CI: 39.5%, 42.8%] of respondents who were not lonely.

### Odds ratios of generalized anxiety by demographics and loneliness status

Table 4 shows the odds of generalized anxiety given all variables, across unadjusted and adjusted models. This section highlights statistically significant results from Model 3, which included all demographic variables in addition to loneliness and clinical diagnosis of PTSD, depression, or anxiety in the past 12 months, unless otherwise indicated. Respondents in the 45–54 age group (OR: 0.56 [95% CI: 0.34, 0.91]) and the 55 or older age group 0.41 [95% CI: 0.22, 0.75] had reduced odds of generalized anxiety compared to those in the 18–24 age group. Women had 1.62 [95% CI: 1.23, 2.15] times the odds of generalized anxiety compared to men. Compared to respondents with higher education, respondents with secondary education had 1.65 [95% CI: 1.18, 2.31] and those with primary education or no education had 2.23 [95% CI: 1.45, 3.42] times the odds of generalized anxiety.

**Table 4:** Logistic regression of generalized anxiety by sample demographics, loneliness status, and past 12-month clinical diagnosis

Characteristic	Model 1: Unadjusted OR [95% CI]	Model 2: Adjusted OR excluding past 12-month clinical diagnosis [95% CI]	Model 3: Adjusted OR including past 12-month clinical diagnosis [95% CI]
**Age group**			
18–24	Reference	Reference	Reference
25–34	0.78 [0.58, 1.05]	0.81 [0.56, 1.15]	0.76 [0.52, 1.11]
35–44	0.61 [0.45, 0.83]**	0.67 [0.45, 1.01]	0.65 [0.42, 1.01]
45–54	0.55 [0.39, 0.78]***	0.61 [0.38, 0.98]*	0.56 [0.34, 0.91]*
55 or older	0.35 [0.23, 0.54]***	0.43 [0.23, 0.79]**	0.41 [0.22, 0.75]**
**Gender**			
Man	Reference	Reference	Reference
Woman	2.09 [1.66, 2.63]***	1.96 [1.51, 2.56]***	1.62 [1.23, 2.15]***
**Educational attainment**			
Higher education	Reference	Reference	Reference
Secondary education	1.71 [1.28, 2.28]***	1.57 [1.13, 2.18]**	1.65 [1.18, 2.31]**
Primary or no education	1.66 [1.19, 2.33]**	2.04 [1.35, 3.08]***	2.23 [1.45, 3.42]***
**Income quintile**			
Highest quintile	Reference	Reference	Reference
Fourth quintile	0.72 [0.46, 1.12]	0.62 [0.39, 0.98]*	0.61 [0.38, 0.98]*
Middle quintile	1.37 [0.90, 2.07]	0.94 [0.61, 1.44]	0.98 [0.63, 1.51]
Second quintile	1.67 [1.11, 2.52]*	1.03 [0.68, 1.56]	0.92 [0.59, 1.42]
Lowest quintile	2.24 [1.50, 3.34]***	1.32 [0.87, 2.01]	1.27 [0.83, 1.94]
**Marital status**			
Married	Reference	Reference	Reference
Never married	2.23 [1.74, 2.87]***	1.17 [0.82, 1.67]	1.04 [0.73, 1.49]
Living with partner	2.77 [1.95, 3.94]***	1.31 [0.86, 1.99]	1.07 [0.70, 1.65]
Divorced or separated	2.07 [1.40, 3.05]***	0.80 [0.50, 1.30]	0.72 [0.44, 1.18]
Widowed	1.10 [0.54, 2.22]	0.74 [0.32, 1.70]	0.75 [0.32, 1.72]
**Urbanicity**			
Large city	Reference	Reference	Reference
Suburb near a large city	1.50 [1.08, 2.08]*	1.95 [1.30, 2.91]**	2.25 [1.50, 3.37]***
Small city or town	1.67 [1.27, 2.18]***	1.82 [1.31, 2.54]***	1.85 [1.31, 2.62]***
Rural area or village	1.19 [0.88, 1.60]	1.62 [1.15, 2.29]**	1.59 [1.11, 2.28]*
**Country**			
United States	Reference	Reference	Reference
Brazil	3.05 [2.10, 4.41]***	2.49 [1.64, 3.78]***	2.64 [1.69, 4.12]***
France	1.04 [0.64, 1.69]	1.01 [0.60, 1.72]	1.00 [0.55, 1.78]
India	0.31 [0.16, 0.62]***	0.37 [0.14, 0.96]*	0.43 [0.17, 1.13]
Indonesia	0.84 [0.52, 1.36]	0.75 [0.42, 1.37]	1.07 [0.58, 1.98]
Nigeria	0.67 [0.40, 1.12]	0.65 [0.36, 1.18]	0.90 [0.49, 1.66]
The Philippines	1.70 [1.11, 2.61]*	1.24 [0.73, 2.08]	1.91 [1.10, 3.31]*
Türkiye	1.16 [0.75, 1.78]	1.46 [0.89, 2.40]	2.01 [1.19, 3.39]**
**Loneliness status**			
No	Reference	Reference	Reference
Yes	6.80 [5.27, 8.77]***	5.65 [4.23, 7.55]***	3.89 [2.86, 5.28]***
**Past 12-month clinical diagnosis** ^1^			
No	Reference	—	Reference
Yes	7.21 [5.76, 9.04]***	—	4.60 [3.46, 6.11]***

p*<0.05; p**<0.01; p***<0.001. The sample was weighted with inverse probability weights forgender and age in CATI countries; gender by age, level of education, employment status, and geographic region in France; and gender by age, race/ethnicity, Census region, metropolitan status, education, and household income in the United States. The models removed 1,587 respondents with missing income, 31 respondents with missing marital status, and 16 respondents with missing urbanicity. Overall, the model removed 1,634 respondents. CI = Confidence Interval, OR = Odds Ratio. Model 1 includes depression and unadjusted covariates (age group, gender, educational attainment, income quintile, marital status, urbanicity, country, loneliness status, and past 12-month clinical diagnosis). Model 2 includes all covariates except for past 12-month clinical diagnosis. Model 3 includes all covariates (age group, gender, educational attainment, income quintile, marital status, urbanicity, country, loneliness status, past 12-month clinical diagnosis).^1^*Past 12-month clinical diagnosis includes diagnosis of post-traumatic stress disorder, depression, or generalized anxiety*

Several associations became weaker between after adjusting for loneliness and past 12-month clinical diagnosis (Model 3). In particular, income-related associations weakened. Compared to the highest income quintile, the odds of generalized anxiety were no longer statistically significant for any income quintile except for the fourth income quintile (OR: 0.61 [95% CI: 0.38, 0.98]). Similarly, the higher odds of generalized anxiety among respondents who were never married, living with a partner, or divorced or separated were no longer statistically significant.

Compared to living in a large city, those living in a suburb near a large city (OR: 2.25 [95% CI: 1.50, 3.37]), a small city or town (OR: 1.85 [95% CI: 1.31, 2.62]), or a rural area or village (OR: 1.59 [95% CI: 1.11, 2.28]) all had higher odds of generalized anxiety. Compared to respondents living in the United States, respondents living in Brazil (OR: 2.64 [95% CI: 1.69, 4.12]), the Philippines (OR: 1.91 [95% CI: 1.10, 3.31]), or Türkiye (OR: 2.01 [95% CI: 1.19, 3.39]) had higher odds of generalized anxiety. Respondents who reported feeling lonely had nearly four times the odds of generalized anxiety compared to those who did not report loneliness, with an odds ratio of (OR: 3.89 [95% CI: 2.86, 5.28]). Having a clinical diagnosis of PTSD, depression, or generalized anxiety in the past 12 months was associated with over four times the odds of generalized anxiety (OR: 4.60 [95% CI: 3.46, 6.11]).

The sensitivity analysis using the GAD-7 ≥ 10 threshold yielded results that were highly consistent with those from the ≥ 15 threshold, with the direction and magnitude of associations largely preserved across demographic and structural factors. As expected with the lower cutoff, several estimates moved toward the null, but a few additional groups—such as adults aged 35–44, widowed respondents, and residents of Nigeria—reached statistical significance only under the ≥ 10 specification. Other predictors, including gender, educational attainment, loneliness, and past-year clinical diagnosis, remained strong and robust across both thresholds. Full sensitivity analysis results for generalized anxiety can be seen in Supplementary Table 4.

Results from the Poisson regression, using GAD-7 symptom scores, were directionally consistent with those from the primary logistic models across all covariates. Differences between models were limited to statistical significance for a small number of covariates. Specifically, the 35–44 age group reached significance only in the Poisson model, and the lowest income quintile showed a persistent elevated risk under Poisson that was attenuated in the fully adjusted logistic model. Select marital status categories demonstrated modest protective associations using the Poisson analysis. At the country level, several countries that were not statistically significant in the logistic models reached significance, though the overall cross-national pattern remained unchanged. Full Poisson regression results for generalized anxiety are presented in Supplementary Table 5 .

## Discussion

A representative, multinational, sample of 7,997 respondents across eight countries found a substantial burden of loneliness coupled with an elevated burden of depression and generalized anxiety across all countries. We also found a strong relationship between loneliness and these two adverse mental health outcomes, although the cross-sectional design cannot establish directionality. The distributions of depression and generalized anxiety in the sample population were patterned by age, gender, educational attainment, and country.

First, our analysis found that nearly four out of ten respondents reported loneliness, a proportion higher than several recent estimates. For instance, a 2023 Gallup survey reported that 20% of adults globally felt lonely, while the Centers for Disease Control and Prevention estimated that 31.9% of US adults experienced social isolation or loneliness in 2022 [10, 40]. These differences may reflect variations in how loneliness is defined and measured, as well as differences in population samples and timing. Nonetheless, the consistently high levels reported across estimates, including our own, underscore the widespread nature of loneliness. Our analysis also showed that nearly one in ten respondents met the criteria for depression and slightly more than one in twenty for generalized anxiety. These results are higher than the WHO global prevalence of 5% for depression and 4% for anxiety disorders (a set of disorders that include generalized anxiety) [41, 42]. The elevated prevalence documented in our study could be due to differences in screening and diagnostic tools, or the effect of the Covid-19 pandemic—or other recent global events—as a mass traumatic event that worsened mental health outcomes over the past few years.

Second, loneliness was consistently and strongly associated with mental health outcomes in our analysis. Respondents who reported feeling lonely had almost three times the odds of depression and nearly four times the odds of generalized anxiety compared to those who did not report loneliness. These findings are consistent with the WHO declaration on loneliness and the US Surgeon General’s advisory [11, 13]. They also align with longitudinal evidence, largely based on data from older populations linking loneliness to depression or anxiety in Denmark, Ireland, and Spain [43–45]. Notably, the study in Ireland also found that baseline anxiety predicted subsequent loneliness, underscoring the bidirectional and reinforcing nature of these associations [44]. Further supporting these findings, a 2021 umbrella review of 13 systematic reviews concluded that loneliness is consistently associated with adverse mental health outcomes, including new-onset depression [46]. Another longitudinal study of adults aged 50 years and older in England found that loneliness was a strong and persistent predictor of depressive symptoms over a 12-year follow-up period, with estimates suggesting that up to 18% of depression cases one year later could be attributed to loneliness [47]. One potential pathway linking loneliness to poor mental health is through diminished social connectedness. Individuals with fewer social ties or less frequent interactions may feel unsupported or disconnected, which can contribute to the onset or worsening of depression and anxiety symptoms [48]. Additional potential pathways include heighted physiological stress reactivity and dysregulation of the hypothalamic–pituitary–adrenal (HPA) axis, which can increase vulnerability to depressive and anxious symptoms [49, 50]. Cognitive mechanisms—such as negative social expectations and increased vigilance to social threat [51]—may also reinforce both loneliness and poor mental health [52]. Behavioral pathways, including sleep disruption [53] and reduced engagement in protective activities [54], may further amplify this relationship. The reverse causal direction is also plausible: symptoms of depression and anxiety may contribute to loneliness by reducing motivation to engage socially, increasing withdrawal, and heightening negative expectations about social interactions [23]. These processes can limit opportunities for connection and reinforce feelings of isolation, further sustaining the cycle between loneliness and poor mental health. The relationship between loneliness and reporting adverse mental health outcomes in our sample remained strong even after controlling for reporting a clinical diagnosis of a mental health condition over the past 12 months.

Third, in addition to loneliness, this analysis showed clear demographic patterns of depression and generalized anxiety, particularly by age, gender, and educational attainment. Depression and generalized anxiety prevalence were highest among respondents aged 18–24 and decreased with age. This is consistent with data from the US, which underscores the heightened vulnerability of younger adults to mental health challenges [55]. Our analysis also found that women bear a higher burden of both depression and generalized anxiety compared to men, consistent with global data [41, 56, 57]. Lower educational attainment was also associated with a burden of depression and generalized anxiety in our analysis. This association between educational attainment and depression is supported by a growing body of research [58–60]. Finally, there were notable differences in the prevalence of depression and generalized anxiety across the surveyed countries. India had the lowest burden and Brazil the highest, with Brazil showing several-fold higher prevalence of both depression and generalized anxiety compared to India.These patterns align with findings from a global meta-analysis of over 60 studies, which reported substantial regional variation in depression and anxiety during the Covid-19 pandemic, with the highest burden observed outside Asia and Europe [61]. Similarly, a multi-country cohort study in Ethiopia, India, Peru, and Vietnam found wide cross-national variation in symptoms of anxiety and depression, mirroring the relative severity of the pandemic [62]. These cross-national differences likely reflect broader variation in demographic composition, economic development, social conditions, and mental health resources across countries, which may shape both the prevalence and reporting of depressive and anxiety symptoms.

### Limitations

This analysis has several limitations. First, our study used screening tools (PHQ-9, GAD-7) to measure depression and generalized anxiety and as such cannot provide clinical diagnosis. However, the screening tools we used have been validated and used across contexts to ensure reliable assessment [29]. Second, loneliness was assessed using a single, binary self-reported item, which does not capture its severity, frequency, or specific dimensions and may introduce misclassification. While this simple emotional-state question aligns with the GSDS’s broad design for consistent, multi-language administration and has demonstrated expected associations in prior applications, we acknowledge the potential for cultural differences in understanding loneliness. Third, response rates were low in several countries (1.1%–16.0%), raising the possibility of nonresponse bias. While weighting cannot fully correct for this, the use of probability-based sampling frames and post-stratification calibration helps reduce demographic imbalance. Moreover, our primary focus is on within-country associations, which are generally less sensitive to response-rate variation than prevalence estimates. Fourth, unmeasured confounding remains possible despite controlling for key factors; however, our models included primary demographic factors identified in prior literature that are also comparable across countries. Finally, the cross-sectional design precludes causal inference on the specific direction of the relationship. However, regardless of whether loneliness causes poor mental health or vice versa, the strong bidirectional associations underscore the urgent need to address both loneliness and mental health challenges simultaneously through integrated public health approaches.

## Conclusion

This cross-sectional, multinational analysis provides contemporary evidence on the substantial burden of loneliness, depression, and generalized anxiety across eight diverse countries. The analysis shows that individuals reporting loneliness experienced higher levels of depressive and generalized anxiety symptoms across demographic groups and national contexts.

While our data cannot establish causality or temporal ordering, the strength and consistency of these relationships highlight the importance of further longitudinal and interventional research to clarify pathways of the relationship between loneliness and adverse mental health outcomes. These robust results align with a broader body of research linking social connection with mental well-being and underscore loneliness as a meaningful public health concern.

## Supplementary Information

Below is the link to the electronic supplementary material.

Supplementary file1

Supplementary file2

## Data Availability

De-identified GSDS data and analytic code are available from the authors upon reasonable request.
