# Supplementary material for: Loneliness, depression, and generalized anxiety across eight countries
Source: Soc Psychiatry Psychiatr Epidemiol. 2026 Feb 5;61(5):909–24. doi: 10.1007/s00127-025-03029-5 (PMC13156131; doi:10.1007/s00127-025-03029-5)
Supplement: Supplementary file 1 — Supplementary file1 [file 127_2025_3029_MOESM1_ESM.docx]

**Supplement Methods**

**S1. Ethical approval process**

The Global Social Determinants of Health Survey was approved by the Boston University Institutional Review Board (H-44020). In addition, data collection in India, Nigeria, and Philippines was approved by local ethics boards, as shown in Table below.

**Table. Local ethics boards and approvals**

| **Country** | **Name of board** | **Reference number** | **Comments** | **Date of approval** |
| --- | --- | --- | --- | --- |
| India | Sigma | 10074/IRB/23-24 | None | 6 January 2024 |
| Nigeria | National Health Research ethics Committee (NHREC) | NHREC/01/01/2007-04/11/2023 | Wording of privacy notice | 6 December 2023 |
| Philippines | Philippines Social Science Council (SSERB) | CF-23-58 | None | 4 January 2024 |

**S2. Pilot**

Ipsos, a leading global market research and public opinion polling company, undertook pilot fieldwork across all countries to ensure adequate sampling approaches, and survey infrastructure and administration. Table below contains information about pilot dates and completed interviews in each country.

**Table. Pilot fieldwork dates and completed interviews**

| Country | Start Date | End Date | Total completed interviews |
| --- | --- | --- | --- |
| Brazil | 25-11-23 | 04-12-23 | 28 |
| Indonesia | 21-11-23 | 04-12-23 | 51 |
| Turkey | 22-11-23 | 27-11-23 | 53 |
| USA | 16-11-23 | 27-11-23 | 50 |
| France | 22-11-23 | 28-11-23 | 72 |
| Nigeria | 19-12-23 | 23-11-23 | 53 |
| Philippines | 12-01-24 | 16-01-24 | 51 |
| India | 16-01-24 | 23-01-24 | 57 |

**S3. Sampling**

Across all eight countries, the target population included adults ages 18 and older residing in the country of interest.

In France and the United States (US), samples were drawn from probability-based online panels that Ipsos maintains in each country. In the US, Ipsos’ KnowledgePanel online panel was generated based on an address-based sampling frame and recruitment done over the years by post mail. In France, the Ipsos’ KnowledgePanel online panel was generated using a dual-frame (mobile and land lines) Random Digit Dialing (RDD) approach, with panelists being recruited via phone. In both countries, the survey was delivered online.

Across the remaining 6 countries, nationally-representative samples were generated using random probability sampling, and Computer-Assisted Telephone Interviewing (CATI) was used to deliver the survey. In these countries, the sampling frame was generated using a Random Digit Dialing (RDD) approach. Mobile phone frames were used, considering they are the predominant device. While a dual-frame approach (mobile and fixed telephone lines) was considered, it was determined to complicate the survey design without enhancing the population coverage.

Sample Solutions, an external sample provider, generated the sample for CATI countries, using the following steps:

1. Identification of mobile phone prefixes in each country.
2. Sample of numbers selected from all numbers using each prefix, ensuring allnnumbers for each prefix had an equal probability of bein selected. Except for India, no statifiaion was used beacuase no meaningful individual information is available on sampling frames. In India, numbers were stratified proportionally by region, given pre-codes can be identified for each region (“telecom circles”).
3. Identification of active numbers using provider lookup query.
4. Provision of sample of active numbers to Ipsos. Sample included (i) phone number, (ii) country, (iii) expected telecommunications provider, and (iv) ported telecommunications provider (if applicable, when user changed provider but kept phone number).
5. Ipsos team checked sample by comparing external market share figures to ensure they matched expected proportions from each provider.

In India, the RDD approach used in the pilot fieldwork demonstated a limitation to gather a sample representative for gender. As a result, upon collecting 550 male interviews, the survey was only delivered when the person answering the phone was female.

While delivering the survey, participants in CATI countries were asked how many working mobile phones/SIM cards they had, to adjust for this variable and have all people have equal selection probability.

**S4. Data collection**

**S4.1. Data collection materials**

Problems pertaining survey length in CATI countries were identified during pilot fieldwork. In addition, some minor question refining was also identified during the piloting process. The survey was reduced in length for CATI countries and questions were refined to give more contect on the purpose of survey; reiterate complete anonymity of response, especially in questions considered sensitive in certain countries; allow for better comprehension; or provide additional response options to better reflect participants’ realities or preferences (e.g. “does not have any savings”). In addition, during the data collection process, a question was added for participants to consent to be recontacted for future rounds of the survey. Only participatns who were asked this question and gave their consent will be eligible for recontact.

The questionnaire was translated to the languages in Table S3 once it had been finalized in English. Translations were reviewed by local teams and fieldwork in each country was not started until the relevant translations had been approved.

**Table. Survey translations by country**

| Country | Language |
| --- | --- |
| Brazil | Portuguese |
| Indonesia | Indonesian |
| Turkey | Turkish |
| USA | English |
| France | French |
| Nigeria | Hausa, Igbo, Yoruba |
| Philippines | Filipino |
| India | Assamese, Bengali, Kannada, Hindi, Gujarati, Tamil, Telugu, Marathi |

To ensure quality of the survey, the Ipsos team performed screen by screen checks of the survey scripted in English against the master questionnaire, as well as the country-specific questions in English against the country-specific versions of the questionnaire, and pre-scripted hard and soft data logic checks.

**S4.2. Data collection teams**

Project managers and interviewers for each country attended training sessions before conducting data collection. In addition to the training, interviewers had to have at least 3 months of experience in social research projects. Further, interviewers also had to participate in role playing and live interviewing activities before beginning pilot data collection. Except in India, refresher trainings were also carried out between pilot and main data collection processes.

**S4.3. Responses**

Table S4 summarizes the sampling method used in each country, as well as the data collection dates and corresponding response rates. Response rates were calculated using the American Association for Public Opinion Research (AAPOR) Response Rate 3 (RR3) and ranged from 1.1% to 16.7%, consistent with contemporary mobile telephone survey performance. Response rates are not reported for the online probability panels in France and the United States (US) because survey invitations go only to active panel members, making telephone-style contact denominators inapplicable. These response-rate differences were addressed analytically through post-stratification weighting (see Section S4).

The response rate in Brazil was lower because it is uncommon to have telephone interviewing and a great number of people did not answer the calls. In Philippines, median length of response was above 30 minutes. As a result, flexibility to slipt the survey across two or more calls was approved, if requested by the participant.

Table. Fieldwork dates and response rates by country

| Country | Mode | Start date | End date | Total Sample Contacted | Total completes | Response rate |
| --- | --- | --- | --- | --- | --- | --- |
| Brazil | CATI | 11 January 2024 | 9 February 2024 | 110,000 | 1,005 | 1% |
| France | Online | 26 January 2024 | 10 February 2024 | — | 1,127 | — |
| India | CATI | 23 January 2024 | 15 February 2024 | 19,250 | 1,001 | 5% |
| Indonesia | CATI | 13 January 2024 | 12 February 2024 | 29,584 | 1,018 | 3% |
| Nigeria | CATI | 11 January 2024 | 30 January 2024 | — | 1,036 | — |
| Philippines | CATI | 19 January 2024 | 17 February 2024 | 33,000 | 1,025 | 3% |
| Turkey | CATI | 12 January 2024 | 12 February 2024 | 23,100 | 1,064 | 5% |
| USA | Online | 16 January 2024 | 28 January 2024 | — | 1,012 | — |

**Note:** For CATI countries, *AAPOR RR3 = completed interviews / (eligible cases + e × unknown cases)*, where *e* is the estimated eligibility proportion among unknown cases.

The survey was expected to take about twenty minutes when delivered over the phone and in English. Across CATI countries, median interview length ranged between 16 to 32 minutes. Table S5 shows the specific interview lengths for each country.

**Table. Interview lengths by country**

| **Country** | **Median (HH:MM:SS)** | **Min**  **(HH:MM:SS)** | **Max**  **(HH:MM:SS)** |
| --- | --- | --- | --- |
| Brazil | 00:21:54 | 00:13:56 | 05:57:57 |
| Indonesia | 00:23:40 | 00:03:36 | 01:59:35 |
| Turkey | 00:22:07 | 00:10:14 | 04:33:25 |
| USA | 00:17:17 | 00:03:52 | 05:52:31 |
| France | 00:19:59 | 00:06:59 | 05:12:28 |
| Nigeria | 00:25:28 | 00:04:01 | 21:35:27 |
| Philippines | 00:32:03 | 00:06:15 | 02:12:47 |
| India | 00:16:43 | 00:05:24 | 05:08:55 |

Each local team checked at least 20-30% of interviews using parallel listening, revieweing of recordings, or back-check calls. Table S6 shows the number of verified interviews in each country. Based on the quality issues identified, specific surveys were cancelled and replaced, or teams were reoriented or retrained.

**Table. Number and proportion of verified interviews by country**

| 1. **Country** | 1. **Total number of interviews completed** | 1. **Number of interviews verified (incl. parallel listening, audio recording and back-checking)** | 1. **% of interviews verified (incl. parallel listening, audio recording and back-checking)** |
| --- | --- | --- | --- |
| 1. Brazil | 1. 1012 | 1. 762 | 1. 75% |
| 1. India | 1. 1027 | 1. 544 | 1. 50% |
| 1. Indonesia | 1. 1018 | 1. 305 | 1. 30% |
| 1. Nigeria | 1. 1032 | 1. 305 | 1. 30% |
| 1. Philippines | 1. 1,017 | 1. 315 | 1. 31% |
| 1. Turkey | 1. 1059 | 1. 256 | 1. 25% |

In CATI countries, and specially in Nigeria and the Philippines, data was skewed towards urban areas, potentially because the sample frame did not cover non-connected population, as well as self-reported data and definitions of what constituted an “urban” and a “rural” area.

Following data collection, checks were conducted to identify impossible or implausible income values. Where such values were found, back-checks were conducted with respondents to obtain correct values, and the dataset was updated accordingly. Country-specific issues were addressed, including recoding ambiguous responses in India (where '1' could mean one lakh/100,000 rupees) and correcting interviewer coding errors in Turkey, where some interviewers keyed “999” when trying to answer with the non-reponse code.

Item non-response was monitored throughout the survey. Respondents could spontaneously refuse any question or respond 'don't know' where applicable, though these options were not read aloud. The survey script required an answer before progression, resulting in no missing data.

Assessment of speeders, to identify respondents who moved very quickly (i.e. three times faster than the mean length of interview) through the survey, was also carried out. Nigeria and India had the highest number of speeders. These interviewes were reviewed and validated by local teams.

Finally, upon finalizing data colletion, Ipsos performed final data quality checks to ensure adequate routing logic, validate response values, identify duplicate records, and assess implausible values and non-response patterns.

**S5. Weighting Procedures**

Weights were constructed in two stages. First, design (base) weights reflected each respondent’s probability of selection. In CATI countries, this probability depended on the number of reachable mobile phone numbers per individual (e.g., individuals with two reachable numbers received half the selection probability of those with one). In the US and France, design weights reflected the probability of selection into the Ipsos KnowledgePanel, accounting for the address-based sampling (US) or dual-frame RDD recruitment (France).

Second, post-stratification calibration was applied to align each country’s sample with national demographic distributions. CATI samples were calibrated to age group and gender. The US and France samples were calibrated to additional margins, including gender by age and education. The US sample was also weighted to race/ethnicity, household income, metropolitan status, and census regions, while the France sample was weighted to employment status and geographic region.

To reduce the influence of extreme weights, design weights for CATI countrieswere trimmed at the 97.5th percentile and final calibrated weights at the 99th percentile. All descriptive and regression analyses in the manuscript applied these final weighted estimates.

**S6. Joint effect of depression and generalized anxiety**

Considering existing evidence that depression and anxiety frequently co-occur, we also examined their joint distribution in our sample. Although our primary models considered depression and generalized anxiety separately, we found that 3.6% (n=315) of adults met criteria for both conditions, indicating meaningful comorbidity. Because this comorbid group was relatively small, we did not fit separate multivariable models for the subgroup with both conditions. See Table below.

**Table. Co-occurrence of depression and generalized anxiety symptoms.**

| **Group** | **Unweighted N = 7,997** | **Weighted % [95% CI]** |
| --- | --- | --- |
| Neither | 7099 | 88.9% [88.1%, 89.7%] |
| Depression only | 441 | 5.6% [5.0%, 6.2%] |
| GAD only | 142 | 1.9% [1.5%, 2.2%] |
| Both depression and GAD | 315 | 3.6% [3.2%, 4.1%] |

**S7. Model Specification**

Survey-weighted regression models were used to estimate associations between loneliness and each mental health outcome. Logistic regression models generated odds ratios (ORs), and modified Poisson models with robust variance produced relative risks (RRs) for sensitivity, given the non-rare prevalence of the outcomes. All models incorporated the final calibrated survey weights.

Three model specifications were estimated for each outcome:

1. Model 1 (Unadjusted): loneliness only.
2. Model 2 (Sociodemographic adjustment): adjusted for age group, gender, education, income quintile, marital status, area of living (urbanicity), and loneliness.
3. Model 3 (Fully adjusted): included all covariates in Model 2 plus past 12-month mental health diagnosis.

Covariates were selected based on established associations with depression, anxiety, and loneliness in prior epidemiologic research and to ensure comparability across countries.

**S8. Model Diagnostics**

Model diagnostics were conducted to evaluate multicollinearity and goodness of fit. Variance inflation factors (VIFs) ranged from 1.02 to 2.18, indicating no meaningful multicollinearity among covariates (Table below). Model fit for the survey-weighted logistic regressions was assessed using design-based likelihood ratio tests implemented via anova.svyglm (Rao–Scott–adjusted). Inclusion of 12-month mental health diagnosis significantly improved model fit for both outcomes: depression (2logLR = 228.7, df = 1, p < 0.001). Likewise, model fit for generalized anxiety disorder improved substantially when loneliness was included (2logLR = 108.0, df = 1, p < 0.001).

**Table. Variance inflation factors (VIFs) assessing multicollinearity in the fully adjusted logistic models**

| **Predictor** | **Degrees of Freedom** | **GVIF (Depression)** | **Adjusted GVIF (Depression)** | **GVIF (Generalized anxiety)** | **Adjusted GVIF (Generalized anxiety)** |
| --- | --- | --- | --- | --- | --- |
| Age group | 4 | 2.17 | 1.1 | 2.17 | 1.1 |
| Gender | 1 | 1.05 | 1.02 | 1.05 | 1.02 |
| Education | 2 | 1.41 | 1.09 | 1.41 | 1.09 |
| Income | 4 | 1.22 | 1.03 | 1.22 | 1.03 |
| Marital status | 4 | 2.01 | 1.09 | 2.01 | 1.09 |
| Urbanicity | 3 | 1.39 | 1.06 | 1.39 | 1.06 |
| Country | 7 | 2.52 | 1.07 | 2.52 | 1.07 |
| Loneliness | 1 | 1.1 | 1.05 | 1.1 | 1.05 |

**S9. Sensitivity Analyses**

Sensitivity analyses were conducted to assess the robustness of findings to alternative symptom thresholds. When depressive symptoms were defined using the standard PHQ-9 ≥10 cutoff instead of ≥15, the weighted prevalence increased from 9.2% (SE = 0.0037) to 20.0% (SE = 0.0052). Agreement between the ≥10 and ≥15 classifications was moderate, with a Cohen’s kappa of 0.58 (95% CI: 0.55–0.60), indicating that the lower threshold identifies additional cases while maintaining consistent classification patterns. Similar results were observed for generalized anxiety symptoms, where applying the GAD-7 ≥10 cutoff increased prevalence and produced moderate agreement with the ≥15 definition (κ ≈ 0.50).

Despite these expected differences in prevalence, the associations between loneliness and both depression and generalized anxiety remained strong, statistically significant, and directionally consistent across both cutoff definitions (Tables below). Effect estimates using the ≥10 thresholds were comparable in magnitude to those obtained using ≥15, demonstrating that the main findings are robust to alternative case definitions. Additionally, models estimated with and without the past 12-month clinical diagnosis variable yielded similar results, indicating that the associations were not driven by prior diagnosis.

**Table. Agreement between PHQ-9 ≥15 and PHQ-9 ≥10 Classifications Using Cohen’s Kappa**

|  | lower | estimate | upper |
| --- | --- | --- | --- |
| unweighted kappa | 0.55 | 0.58 | 0.6 |
| weighted kappa | 0.55 | 0.58 | 0.6 |

**Table. Agreement between GAD-7 ≥15 and GAD-7 ≥10 Classifications Using Cohen’s Kappa**

|  | lower | estimate | upper |
| --- | --- | --- | --- |
| unweighted kappa | 0.48 | 0.5 | 0.53 |
| weighted kappa | 0.48 | 0.5 | 0.53 |

**Table. Prevalence of depression and generalized anxiety by cutoff**

|  | **Weighted Prevalence (95% CI)** | |
| --- | --- | --- |
| Cutoff Threshold | PHQ-9 | GAD-7 |
| ≥ 15 | 9.2% [8.5%, 10.0%] | 5.5% [4.9%, 6.0%] |
| ≥ 10 | 20.0% [19.0%, 21.1%] | 14.9% [14.0%, 15.8%] |
