# Supplementary material for: Loneliness, depression, and generalized anxiety across eight countries
Source: Soc Psychiatry Psychiatr Epidemiol. 2026 Feb 5;61(5):909–24. doi: 10.1007/s00127-025-03029-5 (PMC13156131; doi:10.1007/s00127-025-03029-5)
Supplement: Supplementary file 2 — Supplementary file2 [file 127_2025_3029_MOESM2_ESM.docx]

**Supplementary Tables and Figures**

**Supplementary Table 1: Loneliness status stratified by demographics**

| **Characteristics** | **Total N = 7997** | **Feeling Alone N = 3141** | **Weighted Prevalence [95% CI]** |
| --- | --- | --- | --- |
| **Loneliness status** |  |  |  |
| Yes | 7997 | 3141 | 38.9% [37.6%, 40.2%] |
| **Age group** |  |  |  |
| 18–24 | 1337 | 600 | 46.8% [43.7%, 50.0%] |
| 25–34 | 2074 | 913 | 44.1% [41.7%, 46.5%] |
| 35–44 | 1780 | 711 | 40.0% [37.4%, 42.6%] |
| 45–54 | 1288 | 474 | 35.5% [32.5%, 38.4%] |
| 55 or older | 1518 | 443 | 30.4% [27.5%, 33.3%] |
| **Gender** |  |  |  |
| Man | 4216 | 1558 | 36.6% [34.9%, 38.4%] |
| Woman | 3781 | 1583 | 41.1% [39.2%, 42.9%] |
| **Educational attainment** |  |  |  |
| Higher education | 2530 | 959 | 37.5% [35.2%, 39.7%] |
| Secondary education | 3870 | 1607 | 42.3% [40.5%, 44.2%] |
| Primary or no education | 1597 | 575 | 33.4% [30.7%, 36.2%] |
| **Income quintile** |  |  |  |
| Highest quintile | 1382 | 471 | 34.0% [31.0%, 37.1%] |
| Fourth quintile | 1366 | 471 | 34.2% [31.3%, 37.2%] |
| Middle quintile | 1190 | 467 | 39.9% [36.6%, 43.3%] |
| Second quintile | 1283 | 554 | 42.2% [39.0%, 45.3%] |
| Lowest quintile | 1189 | 589 | 48.1% [44.7%, 51.4%] |
| **Marital status** |  |  |  |
| Married | 4188 | 1255 | 29.5% [27.8%, 31.1%] |
| Never married | 2345 | 1175 | 50.2% [47.8%, 52.5%] |
| Living with partner | 610 | 276 | 45.0% [40.4%, 49.6%] |
| Divorced or separated | 531 | 286 | 53.6% [48.6%, 58.6%] |
| Widowed | 283 | 137 | 47.2% [39.9%, 54.6%] |
| **Urbanicity** |  |  |  |
| Large city | 3000 | 1266 | 41.4% [39.3%, 43.5%] |
| Suburb near a large city | 1164 | 436 | 36.8% [33.5%, 40.0%] |
| Small city or town | 1833 | 719 | 39.2% [36.5%, 41.8%] |
| Rural area or village | 1982 | 713 | 36.0% [33.4%, 38.6%] |
| **Country** |  |  |  |
| United States | 954 | 298 | 31.7% [28.6%, 34.8%] |
| Brazil | 985 | 477 | 47.5% [44.1%, 50.9%] |
| France | 1062 | 375 | 37.4% [33.6%, 41.2%] |
| India | 969 | 214 | 19.2% [16.0%, 22.3%] |
| Indonesia | 1011 | 364 | 36.4% [32.9%, 39.9%] |
| Nigeria | 1021 | 473 | 45.8% [42.0%, 49.7%] |
| The Philippines | 938 | 485 | 48.9% [45.0%, 52.8%] |
| Türkiye | 1057 | 455 | 43.4% [40.0%, 46.8%] |
| **Past 12-month clinical diagnosis** ^1^ |  |  |  |
| No | 6457 | 2095 | 32.5% [31.1%, 33.8%] |
| Yes | 1463 | 1009 | 64.7% [61.9%, 67.5%] |

*Notes: Estimates and confidence intervals weighted using inverse probability weights. Confidence intervals calculated with Rao-Scott correction. The sample was weighted using gender and age in CATI countries; gender by age, education, employment, and region in France; and gender by age, race/ethnicity, Census region, metropolitan status, education, and income in the US. Missing data on loneliness: income quintile (N = 589), past 12-month clinical diagnosis (N= 35), marital status (N = 12), urbanicity (N = 7). ^1^ Past 12-month clinical diagnosis includes diagnosis of post-traumatic stress disorder, depression, or generalized anxiety.*

**Supplementary Table 2: Sensitivity analysis comparing adjusted odds ratios for depression using PHQ-9 ≥15 and ≥10 thresholds**

| **Characteristic** | **Adjusted OR (PHQ-9 ≥15) [95% CI]** | **Adjusted OR (PHQ-9 ≥10) [95% CI]** |
| --- | --- | --- |
| **Age group** |  |  |
| 18–24 | Reference | Reference |
| 25–34 | 0.74 [0.54, 1.00] | 0.85 [0.67, 1.09] |
| 35–44 | 0.54 [0.37, 0.78]** | 0.63 [0.47, 0.83]** |
| 45–54 | 0.47 [0.31, 0.69]*** | 0.65 [0.48, 0.88]** |
| 55 or older | 0.29 [0.18, 0.48]*** | 0.37 [0.26, 0.52]*** |
| **Gender** |  |  |
| Man | Reference | Reference |
| Woman | 1.30 [1.04, 1.61]* | 1.06 [0.90, 1.25] |
| **Educational attainment** |  |  |
| Higher education | Reference | Reference |
| Secondary education | 1.58 [1.21, 2.06]*** | 1.43 [1.17, 1.73]*** |
| Primary or no education | 1.92 [1.34, 2.74]*** | 1.73 [1.33, 2.25]*** |
| **Income quintile** |  |  |
| Highest quintile | Reference | Reference |
| Fourth quintile | 0.89 [0.61, 1.29] | 1.00 [0.77, 1.30] |
| Middle quintile | 1.16 [0.83, 1.63] | 1.15 [0.89, 1.48] |
| Second quintile | 1.15 [0.81, 1.63] | 1.12 [0.87, 1.45] |
| Lowest quintile | 1.51 [1.06, 2.15]* | 1.13 [0.87, 1.47] |
| **Marital status** |  |  |
| Married | Reference | Reference |
| Never married | 0.96 [0.72, 1.28] | 0.99 [0.80, 1.24] |
| Living with partner | 0.79 [0.55, 1.14] | 0.79 [0.60, 1.06] |
| Divorced or separated | 0.84 [0.57, 1.23] | 0.89 [0.65, 1.21] |
| Widowed | 0.85 [0.42, 1.72] | 0.78 [0.46, 1.31] |
| **Urbanicity** |  |  |
| Large city | Reference | Reference |
| Suburb near a large city | 1.50 [1.07, 2.10]* | 1.40 [1.09, 1.80]** |
| Small city or town | 1.39 [1.06, 1.83]* | 1.27 [1.03, 1.56]* |
| Rural area or village | 1.33 [0.98, 1.81] | 1.32 [1.05, 1.65]* |
| **Country** |  |  |
| United States | Reference | Reference |
| Brazil | 1.85 [1.28, 2.70]** | 1.99 [1.48, 2.68]*** |
| France | 1.73 [1.12, 2.65]* | 1.54 [1.13, 2.09]** |
| India | 0.47 [0.23, 0.95]* | 0.70 [0.44, 1.10] |
| Indonesia | 0.63 [0.39, 1.04] | 0.82 [0.58, 1.16] |
| Nigeria | 1.26 [0.82, 1.92] | 1.24 [0.88, 1.74] |
| The Philippines | 1.72 [1.12, 2.65]* | 2.01 [1.46, 2.76]*** |
| Türkiye | 2.02 [1.33, 3.05]*** | 2.95 [2.17, 4.02]*** |
| **Loneliness status** |  |  |
| No | Reference | Reference |
| Yes | 2.82 [2.25, 3.54]*** | 3.54 [3.00, 4.18]*** |
| **Past 12-month clinical diagnosis** ^1^ |  |  |
| No | Reference | Reference |
| Yes | 5.43 [4.35, 6.78]*** | 4.89 [4.09, 5.84]*** |

Notes:*p***<0.05; p****<0.01; p*****<0.001. The sample was weighted with inverse probability weights for gender and age in CATI countries; gender by age, level of education, employment status, and geographic region in France; and gender by age, race/ethnicity, Census region, metropolitan status, education, and household income in the United States.* ^1^ Past 12-month clinical diagnosis includes diagnosis of post-traumatic stress disorder, depression, or generalized anxiety.

**Supplementary Table 3: Poisson regression of depression by sample demographics, loneliness status, and past 12-month clinical diagnosis**

| **Characteristic** | **Model 1: Unadjusted PR [95% CI]** | **Model 2: Adjusted PR Excluding past 12-month clinical diagnosis [95% CI]** | **Model 3: Adjusted PR Including past 12-month clinical diagnosis [95% CI]** |
| --- | --- | --- | --- |
| **Age group** |  |  |  |
| 18–24 | Reference | Reference | Reference |
| 25–34 | 0.94 [0.87, 1.01] | 0.94 [0.88, 1.02] | 0.92 [0.86, 0.99]* |
| 35–44 | 0.83 [0.77, 0.90]*** | 0.84 [0.77, 0.92]*** | 0.84 [0.77, 0.91]*** |
| 45–54 | 0.78 [0.72, 0.85]*** | 0.83 [0.75, 0.91]*** | 0.81 [0.74, 0.89]*** |
| 55 or older | 0.65 [0.59, 0.71]*** | 0.68 [0.61, 0.76]*** | 0.68 [0.61, 0.76]*** |
| **Gender** |  |  |  |
| Man | Reference | Reference | Reference |
| Woman | 1.23 [1.17, 1.30]*** | 1.19 [1.13, 1.25]*** | 1.10 [1.05, 1.16]*** |
| **Educational attainment** |  |  |  |
| Higher education | Reference | Reference | Reference |
| Secondary education | 1.20 [1.14, 1.28]*** | 1.15 [1.08, 1.22]*** | 1.16 [1.09, 1.22]*** |
| Primary or no education | 1.05 [0.97, 1.14] | 1.21 [1.11, 1.32]*** | 1.21 [1.12, 1.32]*** |
| **Income quintile** |  |  |  |
| Highest quintile | Reference | Reference | Reference |
| Fourth quintile | 1.02 [0.93, 1.12] | 0.98 [0.90, 1.06] | 0.99 [0.92, 1.07] |
| Middle quintile | 1.17 [1.07, 1.29]*** | 1.03 [0.95, 1.12] | 1.04 [0.96, 1.12] |
| Second quintile | 1.26 [1.15, 1.38]*** | 1.08 [0.99, 1.17] | 1.07 [1.00, 1.16] |
| Lowest quintile | 1.37 [1.25, 1.51]*** | 1.11 [1.02, 1.21]* | 1.09 [1.00, 1.18]* |
| **Marital status** |  |  |  |
| Married | Reference | Reference | Reference |
| Never married | 1.39 [1.32, 1.48]*** | 1.05 [0.98, 1.12] | 1.02 [0.95, 1.08] |
| Living with partner | 1.36 [1.24, 1.49]*** | 1.04 [0.95, 1.14] | 0.99 [0.91, 1.08] |
| Divorced or separated | 1.37 [1.24, 1.52]*** | 1.05 [0.96, 1.15] | 1.01 [0.93, 1.10] |
| Widowed | 0.98 [0.84, 1.15] | 0.88 [0.75, 1.04] | 0.89 [0.76, 1.03] |
| **Urbanicity** |  |  |  |
| Large city | Reference | Reference | Reference |
| Suburb near a large city | 1.00 [0.92, 1.08] | 1.10 [1.01, 1.19]* | 1.11 [1.03, 1.19]** |
| Small city or town | 1.02 [0.95, 1.09] | 1.06 [0.99, 1.13] | 1.05 [0.98, 1.11] |
| Rural area or village | 0.93 [0.87, 1.00] | 1.07 [0.99, 1.15] | 1.07 [1.00, 1.15] |
| **Country** |  |  |  |
| United States | Reference | Reference | Reference |
| Brazil | 1.63 [1.47, 1.81]*** | 1.43 [1.30, 1.58]*** | 1.38 [1.26, 1.52]*** |
| France | 1.40 [1.26, 1.56]*** | 1.37 [1.24, 1.52]*** | 1.37 [1.25, 1.51]*** |
| India | 0.65 [0.56, 0.77]*** | 0.70 [0.58, 0.85]*** | 0.71 [0.59, 0.86]*** |
| Indonesia | 1.11 [1.00, 1.24] | 0.98 [0.88, 1.09] | 1.08 [0.98, 1.20] |
| Nigeria | 1.21 [1.09, 1.34]*** | 1.19 [1.07, 1.33]** | 1.26 [1.14, 1.40]*** |
| The Philippines | 1.46 [1.31, 1.63]*** | 1.26 [1.13, 1.40]*** | 1.39 [1.26, 1.54]*** |
| Türkiye | 1.68 [1.53, 1.85]*** | 1.60 [1.46, 1.76]*** | 1.72 [1.57, 1.88]*** |
| **Loneliness status** |  |  |  |
| No | Reference | Reference | Reference |
| Yes | 2.22 [2.11, 2.33]*** | 1.97 [1.87, 2.08]*** | 1.69 [1.60, 1.78]*** |
| **Past 12, month clinical diagnosis** *^1^* |  |  |  |
| No | Reference | — | Reference |
| Yes | 2.35 [2.24, 2.46]*** | — | 1.88 [1.78, 1.99]*** |

*Notes: p***<0.05; p****<0.01; p*****<0.001. The sample was weighted with inverse probability weights for gender and age in CATI countries; gender by age, level of education, employment status, and geographic region in France; and gender by age, race/ethnicity, Census region, metropolitan status, education, and household income in the United States. Unadjusted models removed 1,632 respondents with missing income, 43 respondents with missing marital status, and 18 respondents with missing urbanicity. CI = Confidence Interval, PR = Prevalence Ratio. Model 1 assesses depression and unadjusted covariates (age group, gender, educational attainment, income quintile, marital status, urbanicity, country, loneliness status, and past 12-month clinical diagnosis (depression or generalized anxiety)). Model 2 include all covariates except past 12-month clinical diagnosis). Model 3 included all covariates (age group, gender, educational attainment, income quintile, marital status, urbanicity, country, loneliness status and past 12-month clinical diagnosis. ^1^ Past 12-month clinical diagnosis includes diagnosis of post-traumatic stress disorder, depression, or generalized anxiety.*

**Supplementary Table 4: Sensitivity analysis comparing adjusted odds ratios for generalized anxiety using GAD-7 ≥15 and ≥10 thresholds**

| **Characteristic** | **Adjusted OR (GAD-7 ≥15) [95% CI]** | **Adjusted OR (GAD-7 ≥10) [95% CI]** |
| --- | --- | --- |
| **Age group** |  |  |
| 18–24 | Reference | Reference |
| 25–34 | 0.76 [0.52, 1.11] | 0.89 [0.69, 1.16] |
| 35–44 | 0.65 [0.42, 1.01] | 0.65 [0.48, 0.88]** |
| 45–54 | 0.56 [0.34, 0.91]* | 0.48 [0.34, 0.69]*** |
| 55 or older | 0.41 [0.22, 0.75]** | 0.52 [0.36, 0.76]*** |
| **Gender** |  |  |
| Man | Reference | Reference |
| Woman | 1.62 [1.23, 2.15]*** | 1.31 [1.09, 1.57]** |
| **Educational attainment** |  |  |
| Higher education | Reference | Reference |
| Secondary education | 1.65 [1.18, 2.31]** | 1.63 [1.31, 2.03]*** |
| Primary or no education | 2.23 [1.45, 3.42]*** | 2.51 [1.88, 3.35]*** |
| **Income quintile** |  |  |
| Highest quintile | Reference | Reference |
| Fourth quintile | 0.61 [0.38, 0.98]* | 0.65 [0.48, 0.88]** |
| Middle quintile | 0.98 [0.63, 1.51] | 1.08 [0.81, 1.43] |
| Second quintile | 0.92 [0.59, 1.42] | 0.94 [0.71, 1.25] |
| Lowest quintile | 1.27 [0.83, 1.94] | 1.03 [0.78, 1.38] |
| **Marital status** |  |  |
| Married | Reference | Reference |
| Never married | 1.04 [0.73, 1.49] | 0.94 [0.74, 1.19] |
| Living with partner | 1.07 [0.70, 1.65] | 0.92 [0.67, 1.25] |
| Divorced or separated | 0.72 [0.44, 1.18] | 0.72 [0.52, 1.01] |
| Widowed | 0.75 [0.32, 1.72] | 0.44 [0.24, 0.80]** |
| **Urbanicity** |  |  |
| Large city | Reference | Reference |
| Suburb near a large city | 2.25 [1.50, 3.37]*** | 1.37 [1.05, 1.80]* |
| Small city or town | 1.85 [1.31, 2.62]*** | 1.46 [1.16, 1.82]*** |
| Rural area or village | 1.59 [1.11, 2.28]* | 1.28 [0.99, 1.64] |
| **Country** |  |  |
| United States | Reference | Reference |
| Brazil | 2.64 [1.69, 4.12]*** | 2.20 [1.61, 3.01]*** |
| France | 1.0 [0.55, 1.78] | 1.26 [0.87, 1.81] |
| India | 0.43 [0.17, 1.13] | 0.63 [0.36, 1.09] |
| Indonesia | 1.07 [0.58, 1.98] | 0.89 [0.60, 1.30] |
| Nigeria | 0.90 [0.49, 1.66] | 1.47 [1.03, 2.11]* |
| The Philippines | 1.91 [1.10, 3.31]* | 2.22 [1.58, 3.13]*** |
| Türkiye | 2.01 [1.19, 3.39]** | 1.98 [1.42, 2.75]*** |
| **Loneliness status** |  |  |
| No | Reference | Reference |
| Yes | 3.89 [2.86, 5.28]*** | 3.62 [3.01, 4.35]*** |
| **Past 12-month clinical diagnosis** ^1^ |  |  |
| No | Reference | Reference |
| Yes | 4.60 [3.46, 6.11]*** | 4.46 [3.69, 5.40]*** |

Notes:*p***<0.05; p****<0.01; p*****<0.001. The sample was weighted with inverse probability weights for gender and age in CATI countries; gender by age, level of education, employment status, and geographic region in France; and gender by age, race/ethnicity, Census region, metropolitan status, education, and household income in the United States.* ^1^ Past 12-month clinical diagnosis includes diagnosis of post-traumatic stress disorder, depression, or generalized anxiety.

**Supplementary Table 5: Poisson regression of generalized anxiety by sample demographics, loneliness status, and past 12-month clinical diagnosis**

| **Characteristic** | **Model 1: Unadjusted PR [95% CI]** | **Model 2: Adjusted PR Excluding past 12-month clinical diagnosis [95% CI]** | **Model 3: Adjusted PR Including past 12- month clinical diagnosis [95% CI]** |
| --- | --- | --- | --- |
| **Age group** |  |  |  |
| 18–24 | Reference | Reference | Reference |
| 25–34 | 0.96 [0.88, 1.04] | 0.98 [0.90, 1.06] | 0.96 [0.88, 1.04] |
| 35–44 | 0.85 [0.78, 0.93]*** | 0.87 [0.79, 0.96]** | 0.87 [0.79, 0.96]** |
| 45–54 | 0.79 [0.72, 0.87]*** | 0.84 [0.75, 0.93]** | 0.82 [0.74, 0.91]*** |
| 55 or older | 0.65 [0.58, 0.72]*** | 0.74 [0.65, 0.84]*** | 0.74 [0.66, 0.84]*** |
| **Gender** |  |  |  |
| Man | Reference | Reference | Reference |
| Woman | 1.34 [1.26, 1.42]*** | 1.29 [1.22, 1.37]*** | 1.18 [1.11, 1.25]*** |
| **Educational attainment** |  |  |  |
| Higher education | Reference | Reference | Reference |
| Secondary education | 1.23 [1.15, 1.31]*** | 1.19 [1.11, 1.28]*** | 1.20 [1.12, 1.28]*** |
| Primary or no education | 1.11 [1.01, 1.21]* | 1.29 [1.17, 1.43]*** | 1.30 [1.19, 1.43]*** |
| **Income quintile** |  |  |  |
| Highest quintile | Reference | Reference | Reference |
| Fourth quintile | 1.01 [0.91, 1.12] | 0.95 [0.87, 1.05] | 0.96 [0.88, 1.05] |
| Middle quintile | 1.24 [1.12, 1.38]*** | 1.06 [0.96, 1.16] | 1.07 [0.97, 1.17] |
| Second quintile | 1.31 [1.18, 1.46]*** | 1.09 [1.0, 1.20] | 1.08 [0.98, 1.18] |
| Lowest quintile | 1.46 [1.32, 1.62]*** | 1.16 [1.05, 1.28]** | 1.14 [1.03, 1.25]** |
| **Marital status** |  |  |  |
| Married | Reference | Reference | Reference |
| Never married | 1.37 [1.29, 1.47]*** | 1.02 [0.94, 1.10] | 0.98 [0.91, 1.05] |
| Living with partner | 1.45 [1.31, 1.60]*** | 1.06 [0.96, 1.17] | 1.0 [0.90, 1.10] |
| Divorced or separated | 1.33 [1.19, 1.50]*** | 0.92 [0.82, 1.02] | 0.88 [0.80, 0.97]* |
| Widowed | 0.86 [0.70, 1.05] | 0.71 [0.57, 0.87]** | 0.71 [0.59, 0.87]*** |
| **Urbanicity** |  |  |  |
| Large city | Reference | Reference | Reference |
| Suburb near a large city | 1.01 [0.93, 1.11] | 1.10 [1.00, 1.20]* | 1.11 [1.02, 1.21]* |
| Small city or town | 1.07 [0.99, 1.15] | 1.09 [1.01, 1.17]* | 1.08 [1.00, 1.15]* |
| Rural area or village | 0.91 [0.84, 0.99]* | 1.04 [0.96, 1.13] | 1.04 [0.96, 1.12] |
| **Country** |  |  |  |
| United States | Reference | Reference | Reference |
| Brazil | 1.87 [1.67, 2.09]*** | 1.61 [1.45, 1.78]*** | 1.56 [1.42, 1.72]*** |
| France | 1.30 [1.15, 1.46]*** | 1.26 [1.13, 1.41]*** | 1.27 [1.14, 1.41]*** |
| India | 0.55 [0.45, 0.66]*** | 0.61 [0.49, 0.77]*** | 0.63 [0.51, 0.79]*** |
| Indonesia | 0.98 [0.86, 1.12] | 0.91 [0.79, 1.04] | 1.02 [0.90, 1.17] |
| Nigeria | 1.23 [1.09, 1.39]*** | 1.20 [1.06, 1.35]** | 1.29 [1.15, 1.45]*** |
| The Philippines | 1.50 [1.32, 1.69]*** | 1.29 [1.14, 1.46]*** | 1.47 [1.31, 1.64]*** |
| Türkiye | 1.50 [1.34, 1.67]*** | 1.45 [1.30, 1.61]*** | 1.58 [1.43, 1.75]*** |
| **Loneliness status** |  |  |  |
| No | Reference | Reference | Reference |
| Yes | 2.35 [2.23, 2.49]*** | 2.05 [1.93, 2.17]*** | 1.73 [1.63, 1.84]*** |
| **Past 12-month clinical diagnosis** ^1^ |  |  |  |
|  |  |  |  |
| No | Reference | — | Reference |
| Yes | 2.55 [2.42, 2.69]*** | — | 1.98 [1.87, 2.10]*** |

Notes: *p***<0.05; p****<0.01; p*****<0.001. The sample was weighted with inverse probability weights for gender and age in CATI countries; gender by age, level of education, employment status, and geographic region in France; and gender by age, race/ethnicity, Census region, metropolitan status, education, and household income in the United States. The models removed 1,587 respondents with missing income, 31 respondents with missing marital status, and 16 respondents with missing urbanicity. Overall, the model removed 1,634 respondents. CI = Confidence Interval, PR = Prevalence Ratio.*

*Model 1 assesses depression and unadjusted covariates (age group, gender, educational attainment, income quintile, marital status, urbanicity, country, loneliness status, and past 12-month clinical diagnosis (depression or generalized anxiety)). Model 2 include all covariates except past 12-month clinical diagnosis). Model 3 included all covariates (age group, gender, educational attainment, income quintile, marital status, urbanicity, country, loneliness status and past 12-month clinical diagnosis. ^1^ Past 12-month clinical diagnosis includes diagnosis of post-traumatic stress disorder, depression, or generalized anxiety.*

**Supplementary Figure 1: Data inclusion and exclusion flowchart**

Total responses from the survey

(N = 8298)

Eligible respondents

(N = 8015)

Final analytic sample

(N = 7997)

Excluded due to missing age, missing or other gender, or missing or other education

(N = 283)

Excluded due to missing loneliness

(N = 18)
